# What causes mating system shifts in plants? *Arabidopsis lyrata* as a case study: **Updated online 7 December 2016:** This article was originally published under a standard licence, but has now been made available under a CC BY 4.0 licence. The PDF and HTML versions of the paper have been modified accordingly. A corrigendum has also been published.

**DOI:** 10.1038/hdy.2016.99

**Published:** 2016-11-02

**Authors:** B K Mable, J Hagmann, S-T Kim, A Adam, E Kilbride, D Weigel, M Stift

**Affiliations:** 1 Institute of Biodiversity, Animal Health and Comparative Medicine, University of Glasgow, Glasgow, UK; 2 Department of Molecular Biology, Max Planck Institute for Developmental Biology, Tübingen, Germany; 3 Computomics GmbH, Tübingen, Germany; 4 Centre for Genome Engineering, Institute for Basic Science, Daejeon, South Korea; 5 Department of Biology and Ecology, University of Konstanz, Konstanz, Germany

## Abstract

The genetic breakdown of self-incompatibility (SI) and subsequent mating system shifts to inbreeding has intrigued evolutionary geneticists for decades. Most of our knowledge is derived from interspecific comparisons between inbreeding species and their outcrossing relatives, where inferences may be confounded by secondary mutations that arose after the initial loss of SI. Here, we study an intraspecific breakdown of SI and its consequences in North American *Arabidopsis lyrata* to test whether: (1) particular *S*-locus haplotypes are associated with the loss of SI and/or the shift to inbreeding; (2) a population bottleneck may have played a role in driving the transition to inbreeding; and (3) the mutation(s) underlying the loss of SI are likely to have occurred at the *S*-locus. Combining multiple approaches for genotyping, we found that outcrossing populations on average harbour 5 to 9 *S*-locus receptor kinase (SRK) alleles, but only two, S1 and S19, are shared by most inbreeding populations. Self-compatibility (SC) behaved genetically as a recessive trait, as expected from a loss-of-function mutation. Bulked segregant analysis in SC × SI F2 individuals using deep sequencing confirmed that all SC plants were S1 homozygotes but not all S1 homozygotes were SC. This was also revealed in population surveys, where only a few S1 homozygotes were SC. Together with crossing data, this suggests that there is a recessive factor that causes SC that is physically unlinked to the *S*-locus. Overall, our results emphasise the value of combining classical genetics with advanced sequencing approaches to resolve long outstanding questions in evolutionary biology.

## Introduction

Uncovering the mechanisms regulating genetically controlled self-incompatibility (SI) systems in plants and fungi has been of sustained interest to the Genetics Society research community, with articles since the inception of *Heredity* (see, for example, Lewis, 1947; Bateman, 1952). A search for ‘incompatibility' in *Heredity* archives retrieved 969 publications, with 275 related specifically to reproductive systems. Nevertheless, there is much that we still do not understand. SI is widespread and has multiple independent origins throughout the plant kingdom (see, for example, Raduski *et al.*, 2012). However, it has proven difficult to explain how these recognition systems that require paired specificity of male and female components evolve and are maintained (Charlesworth, 1988, 1995). A shift from outcrossing to inbreeding is one of the most frequent evolutionary transitions in plants (reviewed in Igic *et al.*, 2008). Nevertheless, what causes breakdown of genetically controlled SI systems and how inbreeding lineages can evolve in the face of inbreeding depression remains poorly understood (reviewed by Vekemans *et al.*, 2014). The rapid technological advances of the past two decades offer new possibilities to address the possible drivers and genetic bases of these transitions.

The Brassicaceae (mustard family) have emerged as a model system for investigating the breakdown of SI. Key to the SI response is the recognition of self-pollen conferred by the *S*-locus receptor kinase (SRK) protein expressed on the stigma (Stein *et al.*, 1996) that has a matching protein (*S*-locus cysteine rich or *S*-locus protein 11 (SCR/SP11); Schopfer *et al.*, 1999) expressed in the pollen coat. Pollen grains that express a variant of SCR matching that of the SRK expressed on the receiving stigma are rejected. The genes encoding these female and male proteins are physically linked and form the *S-*locus, which is found in a genomic region that shows restricted recombination between a U-box domain protein (At4g21350; B80) and a member of the *SRK* gene family (*ARK*3) (Goubet *et al.*, 2012; Roux *et al.*, 2013). There is a complex downstream signalling reaction that is still not completely understood (Goring, 2000; Iwano *et al.*, 2015), but self-compatibility (SC) species typically lack activity of some of these downstream components (for example, Arm-Repeat-Containing Protein 1 (ARC1)) (Indriolo *et al.*, 2012). The SI in Brassicaceae is sporophytic, meaning that expression of both male and female components can be affected by dominance interactions because the protein on the surface of the pollen is deposited by the diploid anther cells (Hatakeyama *et al.*, 1998).

The ancestral state of sporophytic SI (see, for example, Igic *et al.*, 2008) has broken down in several Brassicaceae lineages, and has given rise to highly selfing species. This transition involves a two-step process: loss of SI at the level of individuals, followed by a shift to inbreeding at the population level (see Haudry *et al.*, 2012). Theory predicts that ecological factors such as mate limitation favour inbreeding (Byers and Meagher, 1992; Vekemans *et al.*, 1998); for example, at the front of colonisation waves at range edges (Baker's law; Baker, 1955; Pannell *et al.*, 2015). *S*-allele diversity is usually much reduced in inbreeding lineages (reviewed by Vekemans *et al.*, 2014), but it is typically difficult to deduce whether the shift to inbreeding occurred as a result of mate limitation due to a bottleneck in *S*-alleles, or whether the bottleneck in *S*-alleles was a result of a selective sweep for self-fertilisation, combined with inherently reduced effective population size in highly inbred populations (Glemin *et al.*, 2006).

Unravelling the mechanisms that originally caused functional loss of SI has posed a substantial challenge, partly because most comparisons so far have been made between species where other transitions such as changes in floral morphology and life history strategies could confound interpretations. Theoretically, loss of SI could be caused by: (1) recombination at the *S-*locus that breaks up paired specificity of male and female components; (2) mutations in either the female or male recognition genes that cause a loss of function or lack of recognition; (3) modifiers that affect the expression of *S*-alleles; or (4) mutations in unlinked genes required for the downstream incompatibility response. Conclusions based on transitions occurring in highly selfing *Arabidopsis thaliana* or comparisons with its outcrossing relatives have yielded conflicting conclusions (Indriolo *et al.*, 2012; Nasrallah and Nasrallah, 2014; Vekemans *et al.*, 2014; Shimizu and Tsuchimatsu, 2015). Investigating the causes and consequences of loss of SI and a shift to inbreeding within a species that both shows variation in outcrossing rates among populations and still segregates for SC within outcrossing populations could yield new insights. Such an approach should help to disentangle mechanisms for loss of SI from subsequent changes occurring once inbreeding has become established.

*Arabidopsis lyrata* provides such a model: it is a largely SI relative of *A. thaliana*, but in the Great Lakes region of eastern North America, multiple populations have become predominantly inbreeding and a breakdown of SI is observed even in individuals from highly outcrossing populations (Mable *et al.*, 2005; Mable and Adam, 2007). A previous study comparing *S*-locus genotypes of SC and SI individuals of *A. lyrata* from this region failed to identify an association with particular *S*-haplotypes (Mable and Adam, 2007), suggesting that the mechanistic causes of loss of SI might be different in *A. lyrata* compared with other Brassicaceae (Vekemans *et al.*, 2014). However, the previous study was limited for two reasons. First, only two highly inbreeding populations were included that later turned out to be from different population genetic clusters and thus may represent independent shifts to inbreeding (Foxe *et al.*, 2010). Second, reliable identification of *S*-alleles is challenging because of their high divergence (Mable *et al.*, 2003; Schierup *et al.*, 2006; Mable and Adam, 2007; Schierup *et al.*, 2008), which impeded accurate comparisons of *S*-locus diversity between inbreeding and outcrossing populations. Advances in sequencing technology and characterisation of *S*-locus genomic regions from multiple *S*-haplotypes (Goubet *et al.*, 2012) now make it possible to perform a broader survey of *S*-haplotype variation and to conduct a detailed assessment of the mechanisms of loss of SI.

The purpose of this study was to investigate the cause of loss of SI and subsequent shift to inbreeding within a species where populations that differ in mating system are found in close geographic proximity, using a combination of classical and newer deep sequencing approaches. Specifically, we compared patterns of *S*-locus variation in inbreeding and outcrossing populations of *A. lyrata* from the Great Lakes region and predicted the number of *S*-haplotypes segregating in these populations. We then performed a bulked segregant analysis using short-read sequencing of pools of individuals segregating for SC in F_2_ progeny of experimental crosses, in order to identify the genomic regions that differ between SC and SI pools. This allowed us to test whether: (1) the loss of SI and/or the shift to inbreeding in *A. lyrata* is associated with particular *S*-haplotypes; (2) an *S*-locus bottleneck may have played a role in driving the transition to selfing; and (3) loss of SI is due to mutations at the *S*-locus, modifiers of the recognition response or mutations in downstream components of the signalling pathway.

## Materials and methods

### Study system

To screen variation at *SRK* and flanking genes, we used DNA samples extracted from 192 individuals from 24 populations (8 individuals per population) with known breeding and mating system (Foxe *et al.*, 2010): 16 populations were predominantly SI and outcrossing (0.6<*T*_m_<0.99), 7 were predominantly SC and inbreeding (0<*T*_m_<0.40) and 1 was classified as mixed mating, based both on an intermediate outcrossing rate (*T*_m_=0.41) and the equal presence of both SI and SC individuals (Supplementary Table S1).

### Characterisation of the *S*-locus in inbreeding and outcrossing populations

#### SRK genotyping

To compare variation among inbreeding and outcrossing populations at genes directly involved in SI, we focussed on the female component (*SRK*), because the male component (*SCR*) has not been sufficiently characterised to allow effective screening of large numbers of samples. We initially used allele-specific forward primers targeting seven *SRK* alleles previously found in the Great Lakes populations (S_1_, S_3_, S_13_, S_19_, S_20_, S_23_ and S_39_) with a general reverse primer (*SLGR*; see Supplementary Table S2). These primers were selected because of their consistent amplification of *SRK* alleles fully linked to the SI response, and with known dominance relationships (Schierup *et al.*, 2001; Prigoda *et al.*, 2005; Mable and Adam, 2007). For clarity, we use ‘allele' to refer to variants at particular genes within the *S*-locus and ‘haplotype' to refer to the specificity conferred by the combination of male and female components, along with their associated flanking genes.

We then complemented this partial genotyping by cloning and sequencing *SRK* amplicons from a subset of individuals from each population, using three sets of degenerate forward primers (13FBM, 13-3sF and *SRK* 497F) with *SLGR* (see Supplementary Table S2 and Supplementary Information). We also piloted a long-read tagged amplicon approach using MiSeq (Illumina, San Diego, CA, USA) on 24 samples (3 individuals from each of HDC, IND, MAN, PCR, PIN, SBD, TSSA and TSS; see Supplementary Information). Briefly, the method allowed sequencing of 900 bp products by shearing of barcoded amplicons. For each sample, CLC Genomics workbench (version 7.5, Qiagen Aarhus, Aarhus, Denmark) was used to assemble contigs *de novo* and map paired reads back onto them (see details in Supplementary Information). The consensus sequences were then extracted and BLAST was used to identify the most similar sequences available in GenBank.

#### Characterisation of *S*-locus haplotypes

To assess whether inbreeding and outcrossing populations also differed in broader *S*-haplotypes, we sequenced several genes flanking the *S*-locus: B160 (transcription factor; At4g21430) and B120 (*S*-locus lectin kinase 9; At4g21390) are upstream of the recognition genes *SRK/SCR*, whereas B80 (U-box domain protein; At4g21350) and B70 (Ethylene-responsive protein-like transcription factor; At4g21340) are downstream. For all individuals in our study, B80 and B160 had been sequenced previously (Haudry *et al.*, 2012; Popset accessions: 374282218 and 374282986); here we used primers developed by Kamau and Charlesworth (2005) to sequence and genotype B70 and B120. Strategies for direct sequencing, cloning and haplotype resolution were as described in Haudry *et al.* (2012).

We then tested whether different flanking variants associated with the same *SRK* allele were monophyletic (that is, suggesting common origins) or whether patterns of variation were more consistent with the geographic distribution or mating system of the sampled populations, by reconstructing genealogies for each gene using MEGA 6.0 (Tamura *et al.*, 2013). After applying Model Test (as implemented in MEGA) to choose the most appropriate model of evolution, we performed a maximum likelihood analysis with 1000 bootstrap replicates. We then mapped *SRK* variants, individual populations and genetic clusters that had been inferred from STRUCTURE analysis of microsatellite loci (Foxe *et al.*, 2010) onto these trees. In addition, we used individuals that were homozygous at *SRK* and B80 to assess whether inbreeding populations shared unique *S*-locus haplotypes (that is, based on *SRK* and the four flanking genes) or represented a subset of the diversity found in outcrossing populations.

### Estimating the number of *S*-haplotypes within populations

Given previous evidence of strong linkage disequilibrium between B80 and the *S*-locus in *A. lyrata* (Hagenblad *et al.*, 2006; Kamau *et al.*, 2007), we used heterozygosity at this locus to predict when we had likely missed alleles at *SRK* in order to estimate the number of *S*-alleles in each population. We used the genealogies to predict cases where particular *SRK* variants were associated with more than one B80 allele or where different *SRK* variants appeared to share a B80 allele. This was taken into account in the prediction of heterozygosity.

Here, we used the repeatability index of Stevens and Kay (1989), which provides meaningful estimates for sporophytic SI systems that can have unequal allele frequencies because of dominance (Mable *et al.*, 2003). We calculated the predicted number of *S*-haplotypes in each population using the formula *N*=1−[(*n*−2)/(*m*−2)], where *N* is the number of alleles in the population, *n* is the number of alleles identified in the sample and *m* is the number of gene copies sampled. We calculated the maximum number of alleles assuming every individual within a population had a unique missing haplotype and the minimum assuming they shared a single variant that could not be identified with the methods used.

### Genetic basis of loss of SI

#### Inheritance of selfing phenotype in F_1_ progeny from crosses between SI and SC plants and between SI plants

To study the inheritance of selfing phenotypes, we performed several crosses between plants with known selfing properties: (1) within population crosses between SI plants from two outcrossing populations (MAN and PIN); (2) between SI plants from the MAN population and SC plants from the predominantly selfing PTP population; and (3) between SI plants from the PIN population and SC plants from the predominantly selfing RON population. In all cases, an SI individual was used as recipient (mother) to reduce risk of contamination with pollen from the cross recipient. Subsequently, we determined the selfing phenotype of all F_1_ progeny by performing at least six self-pollinations and scoring fruit set. Plants were considered SC if they produced at least five full siliques in six replicate self pollinations, SI if at least five siliques contained no seeds and leaky SI if two or more siliques showed partial development (Stift *et al.*, 2013).

#### Generation of an F_2_ family that segregates for selfing phenotype

To investigate the genetic basis for the loss of SI, we made use of the F_1_ family derived from the PIN × RON cross, in which all progeny were SI without evidence for leakiness (*n*=20) and for which the parents had been genotyped for the *S*-locus. The PIN parent (PIN 12-3) carried S_23_ and an unknown *SRK* allele S_x_, whereas the RON plant (RON 19-3) had been inferred to be homozygous S_1_S_1_, so that the resulting F_1_ progeny were either S_1_S_x_ or S_1_S_23_. Owing to the recessivity of S_1_ to all other *S*-haplotypes, the S_1_S_x_ and S_1_S_23_ siblings express different specificities (S_x_ and S_23_, respectively) and could thus be crossed to generate biparentally inbred F_2_ progeny (Stift *et al.*, 2013).

To determine segregation of the selfing phenotypes, we raised 97 of these individuals from four S_1_S_x_–S_1_S_23_ F_1_ sibling pairs (Supplementary Table S3). Following the procedures described for the F_1_, most F_2_ plants could be unambiguously grouped into the previously defined classes SC, SI and leaky SI, but male sterility emerged as a fourth phenotype characterised by shrivelled anthers that produced no visible pollen.

To test segregation of *SRK* in the F2 progeny, we originally screened a subset of individuals using allele-specific PCR and sequencing of the alleles present in the grandparents. However, as we were not able to identify one of the alleles (S_x_), we could not distinguish S_1_ homozygotes from S_1_S_x_ heterozygotes using this approach. We thus exploited the linkage disequilibrium of B80 to *SRK* to infer segregation of the *S*-haplotypes in the F_2_ progeny, which was possible because all four genotypes could be resolved by direct sequencing.

#### Bulked segregant analysis (Illumina sequencing)

High-quality DNA extracts were prepared from pools of individuals with the same phenotype (SC or SI) within the F_2_ progeny (see details in Supplementary Information). The SI and SC pools were processed to make sequencing libraries using manufacturer's protocols for whole-genome sequencing on an Illumina GAII instrument. Three lanes of separate runs (two 150 bp and one 100 bp paired-end read run) were sequenced for each pool, resulting in ∼30 Gb of sequence for each pool. The Illumina quality-filtered reads were mapped against the reference genome sequence MN47 (Hu *et al.*, 2011) using GenomeMapper (Schneeberger *et al.*, 2009a), allowing for up to 10% mismatches/gaps relative to the read length. All alternative alleles relative to the reference base with a minimum frequency within each pool of 10% and a score of at least 25 were called by SHORE, as previously described (Ossowski *et al.*, 2008).

#### Identification of SNP sharing among SC and SI pools and SC reference genome sequence

To identify larger genomic regions of different allele frequencies (that is, proportion of reads for each variant found at a single single-nucleotide polymorphism (SNP) site) of genetic variants between the two pools, we employed a strategy similar to the SHOREmap approach (Schneeberger *et al.*, 2009b). Allele frequencies of single positions were then averaged in sliding windows (step size of 10 000 and a window size of 2 00 000 bp) along the genome to yield detectable distinct patterns. The assumption for the SHORE map approach was that genomic regions associated with a particular phenotype should show a depression of heterozygosity in pools of individuals sharing that phenotype as compared with pools of individuals with a different phenotype. To increase the potential strength of this signal, the SNP calls from each pool were compared with a reference genome obtained from a SC individual from the same highly inbreeding population as the SC parent used for the crosses. Given the high heterozygosity expected for *A. lyrata*, this reference was produced by crossing individuals from RON (sampled from Rondeau Provincial Park in Ontario) to plants raised from seeds from the inbred line of the *A. lyrata* reference genome, MN47 (Hu *et al.*, 2011), which was from one of the outcrossing populations that we used for our *SRK* survey (IND; from Indiana Dunes National Lakeshore in Michigan) (see details in Supplementary Information). We predicted that genomic regions associated with the loss of SI would show sharing between the SC pool and the AL4 reference, whereas the SI pool would be polymorphic or show different mutations in these regions. We used the SHOREmap sliding window analysis to identify broad chromosomal regions showing an excess of homozygosity in the SC pools shared with the AL4 reference and then compared individual SNP calls in these regions to identify particular genes or regions that also showed allele sharing between the SC pools and the AL4 reference but not the SI pool. We then extracted the consensus sequences and used BLAST to ascertain the identity of any genes found in such regions.

#### *S*-locus characterisation in SC and SI pools

To specifically determine whether there were differences at the *S*-locus between the SC and SI pools, we used a sequence- rather than a SNP-based approach, where we could take advantage of the known haplotype structure of S_1_ based on a previous bacterial artificial chromosome (BAC) study (Goubet *et al.*, 2012) and our own flanking gene sequencing. The MN47 reference strain is known to have an S_13_ haplotype (Hu *et al.*, 2011) that shows only 71% similarity (in the extracellular *S*-domain) to the S_1_ and S_23_ haplotypes expected in the pools and hence should be clearly distinguishable. The genomic structure of S_23_ has not been resolved but we downloaded sequences for five flanking genes (B160, B120, *ARK*3, B80 and B70), *SRK* and *SCR* from the published BAC sequence for the S_1_ genomic region (accession numbers: KJ772401–4) to use as references, along with an *SRK* sequence for S_23_ obtained from our population survey. We extracted the consensus sequences of the SI and SC pools using SAMtools (Li, 2011), with IUPAC (The International Union of Pure and Applied Chemistry) ambiguity codes used to indicate heterozygous and homozygous sites.

We used CLC Genomics Workbench (CLC, Aarhus, Denmark) to map the following to the SI and SC consensus sequences: (1) the reference sequences for *SRK*, *SCR* and the flanking genes; and (2) other members of the *SRK* gene family that are not linked to the SI phenotype (*Aly*7, *Aly*9, 13-2, *ARK1* and *ARK2*, Charlesworth *et al.*, 2003; accession numbers: AY186754, AY186756, AY186763, AY186758 and AY186761). We also mapped published sequences from *A. lyrata* for one of the downstream components of the SI signalling cascade that has been implicated in loss of SI (*ARC1*; accession number: KF418158.1) (Indriolo *et al.*, 2014), along with another member of that gene family whose relationship to SI remains unclear (*PUB17*: accession number: XM_002890762.1) (Hu *et al.*, 2011). We used the consensus sequences for each gene targeted to determine whether the SI or SC pools differed in heterozygosity or sequence polymorphism. We predicted that if loss of SI was associated with the *S*-locus itself, then genes at the *S*-locus should show a difference between SI and SC pools, whereas unlinked members of the *SRK* gene family should not. We also searched the unmapped and raw reads for each of the reference sequences including the three B80 variants segregating in the crosses in order to predict the *SRK* alleles present in each of the two pools.

## Results

### Characterisation of *S*-haplotypes in inbreeding and outcrossing populations

Allele-specific screening revealed all seven alleles previously known to occur in the Great Lakes area among the outcrossing populations, with the inbreeding populations having only three (Table 1 and Supplementary Table S4). Cloning using degenerate primers did not yield further information, as other members of the gene family preferentially amplified (Supplementary Table S4). However, the MiSeq analysis of 24 outcrossing individuals appeared promising (see Supplementary Information for more details); alleles identified through allele-specific PCR could always be confirmed with the MiSeq analysis and more heterozygotes were resolved using the latter (Supplementary Table S5). This analysis also identified an additional allele known to be linked to the SI phenotype (S_27_) and one putatively new allele (named AlySRK52, 80% similar to AlySRK15).

The 48 individuals from predominantly inbreeding populations all had *SRK* allele S_1_, S_19_ or both. Of these, 16 individuals only showed the presence of S_1_ (all the individuals from the RON and PTP populations), 27 only amplified S_19_ (from the remaining inbreeding populations) and 4 were S_1_S_19_ heterozygotes (all from the WAS population). The TC population also contained one S_3_S_19_ heterozygote. The mixed mating population TSSA contained S_1_ and S_19_, but also S_3_, S_13_ and S_27_.

**Figure 1:**
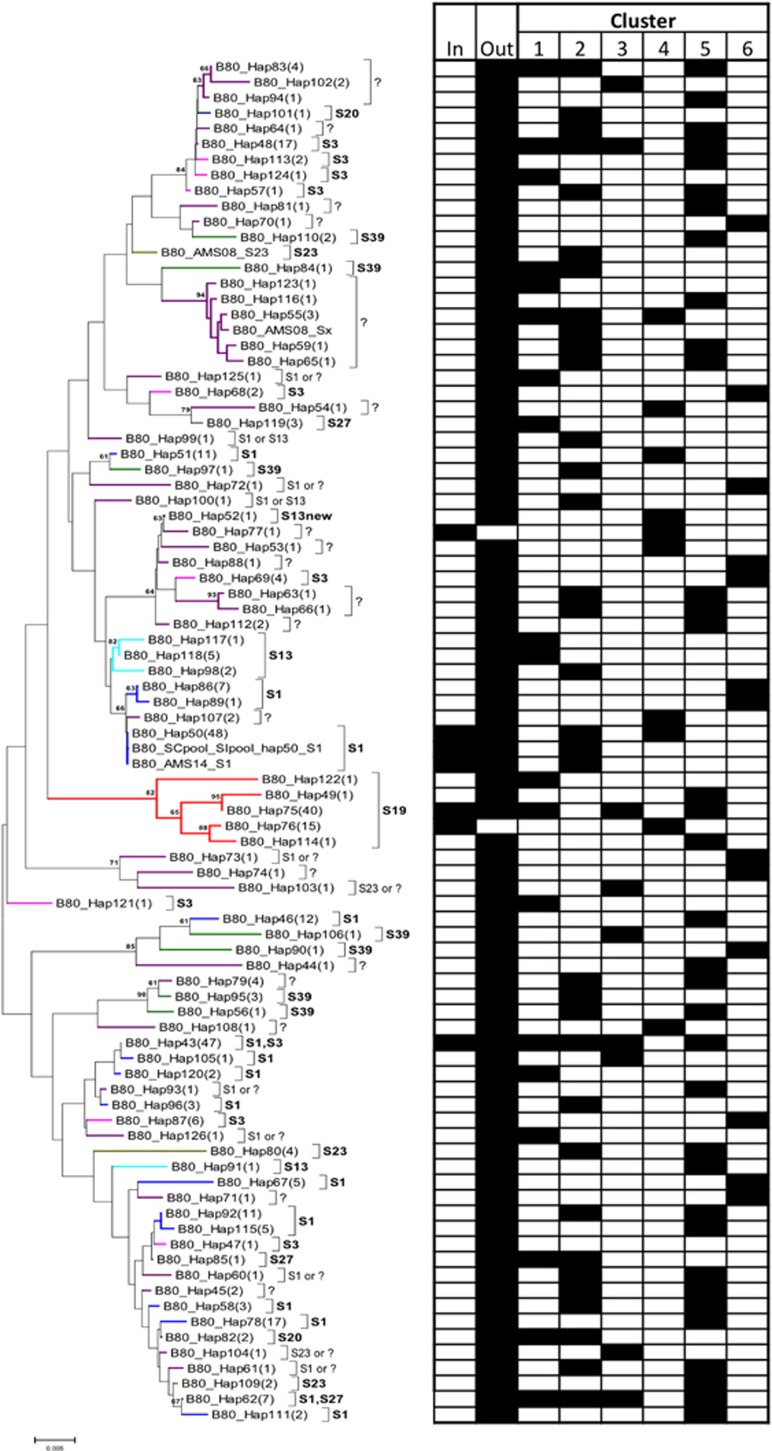
Minimum evolution genealogy of B80 alleles, indicating associations with *SRK* alleles and geographic distribution. The frequency of each allele is indicated in parentheses after its name. The tree was reconstructed using MEGA 6.0, under a Kimura 2 parameter model of evolution, with rate heterogeneity modelled under a gamma distribution using a rate parameter of 0.45. Numbers on the nodes indicate bootstrap support based on 1000 pseudoreplicates. As low phylogenetic resolution is expected for genes evolving under balancing selection, the main purpose of the tree is for visualisation of relatedness among B80 alleles in relation to their association with *SRK* alleles. Associated *SRK* alleles are indicated by name and using coloured branches. Occurrences of each B80 allele in inbreeding and outcrossing populations and in each of the six genetic clusters predicted by STRUCTURE are indicated in the table to the right.

Heterozygosity at B80 suggested that an additional unidentified *SRK* allele was present in one of the individuals from LPT, with all other individuals from inbreeding populations where only one *SRK* allele was amplified being homozygous at B80 (Supplementary Table S4). All of the inbreeding populations with S_19_ shared one of two B80 variants (which differed at 5 out of 666 bp): hap75, which was found in most of the populations, and hap76, which was only found in LPT (Table 2 and Supplementary Table S4). All individuals from the LPT population shared a single synonymous mutation in the *S*-domain region of S_19_. B80 hap75 was found in SI individuals from outcrossing populations but outcrossing populations also had other variants (haps 49, 114 and 122). Although the other flanking genes in S_19_ homozygotes showed more variation, the LPT population also had unique variants of B120 and B160 that were absent from the other inbreeding populations and the outcrossing populations (Table 2 and Supplementary Table S4). As S_19_ is a dominant allele, homozygotes were absent from the outcrossing populations but B80 hap75 was also found in an outcrossing population (PUK; Supplementary Table S4). The genealogy suggested that the B80 haplotypes associated with S_19_ were monophyletic (Figure 1), whereas those associated with other alleles were not (Supplementary Figures S1–S3 and Supplementary Table S6).

The recessive allele S_1_ was found at high frequency among the outcrossing populations and was associated with B80 haplotypes distributed across the genealogy. However, the inbreeding populations contained only two B80 haplotypes (Figure 1 and Supplementary Table S4): hap50 was shared only with outcrossing populations in geographic proximity to inbreeding populations (HDC and PRI), whereas hap43 was widespread among outcrossing populations from different regions. There was also only a single B120 haplotype in RON and PTP that was shared with HDC and PRI.

### Estimating the number of *S*-haplotypes within populations

The MiSeq analysis resolved complete heterozygous *SRK* genotypes for 16/24 of the samples screened, and identified one new putative *SRK* allele. Six individuals were predicted to be homozygous and three were predicted to have an unidentified *SRK* allele, based on heterozygosity at B80 (Supplementary Tables S4 and S5). *ARK*3 (*Aly*8) was not as reliable as B80 for predicting heterozygosity, as in some cases homozygotes for *ARK*3 had two *SRK* alleles in the MiSeq analysis (Supplementary Table S5 and Supplementary Information).

Some individuals for which only S_1_ was amplified were heterozygous at B80 for two different alleles associated with S_1_ (Supplementary Table S4) from disparate parts of the tree (Figure 1). These individuals were thus hypothesised to be homozygous for S_1_ but originating from two different genetic backgrounds; results are also presented assuming that the *SRK* alleles were not the same (Supplementary Table S7). Based on the repeatability index of Stevens and Kay (1989), outcrossing populations were predicted to have on average between 5 and 9 *S-*haplotypes per population, whereas inbreeding populations were predicted to have 1.4 (Table 1 and Supplementary Table S7). There was little difference in the number of *S*-haplotypes predicted in different clusters.

### Inheritance of the selfing phenotype

Although most F_1_ progeny from the within-population crosses involving SI individuals from the outcrossing population MAN yielded SI individuals, one individual (out of 28 screened) was SC, and leaky SI was found in all of the families (Supplementary Figure S4). Crosses between SI and SC plants (MAN × PTP) yielded a mixture of SC and SI phenotypes (20 SC out of 71 screened). All F_1_ progeny from crosses involving the outcrossing PIN were SI but the 97 F_2_ progeny from a cross between an SI individual from PIN and a selfing individual from RON segregated for the selfing phenotype: 10 were SC; 71 were SI; 4 were leaky SI; 12 were male sterile; and self-pollinations gave ambiguous results for one. For a balanced comparison in the bulked segregant analysis, we thus combined DNA extracted from all 10 SC individuals for the SC pool and 10 SI individuals for the SI pool. The SI individuals were selected from across the four families (Supplementary Tables S3 and S8).

### The genetic basis of loss of SI

In the F_2_ progeny segregating for the selfing phenotype, *S*-haplotype segregation based on B80 genotypes revealed that all SC individuals were homozygous for S_1_, whereas the SI phenotypes included the 3 heterozygous combinations and a single S_1_ homozygote (Supplementary Table S3). There was also complete correspondence between genotypes based on direct *SRK* sequencing and B80 sequencing (Supplementary Table S3). However, there was evidence for a segregation bias that differed among the four families pooled for the bulked segregant analysis (Supplementary Table S8). Two families showed a significant deficit of S_1_S_1_ and S_1_S_x_ genotypes and a deficit of S_1_ alleles overall; one of these families produced exclusively SI individuals, whereas the other showed 10% SC individuals and included 30% of individuals with a male sterile phenotype. The remaining two families showed no bias in terms of genotypes or alleles; one had 14% SC and 21% male sterile individuals, whereas the other had 14% SC individuals but did not include any that were male sterile.

**Figure 2:**
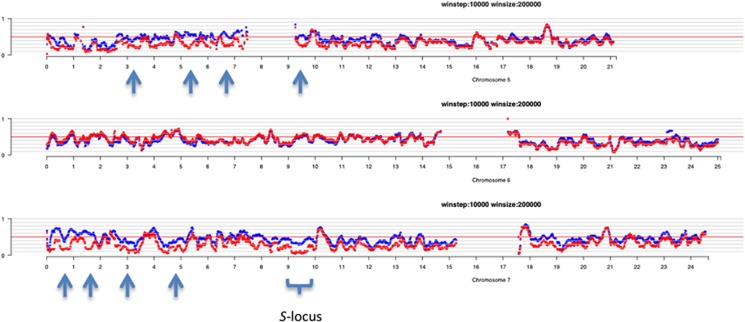
SHOREmap output for chromosomes 5, 6 and 7. The trace in red shows comparison of the SC pool with the reference sequence AL4 (from an SC individual from RON) and the trace in blue shows that for the SI pool. The scale at the bottom shows the position along the chromosome. The plots were produced using a step size of 10 000 and a window size of 200 000 bp. For each chromosome plot, the y axis indicates the proportion of reads either matching or showing an alternative to the reference sequence: 0 indicates fixation of variants that match AL4 and 1 indicates fixation for a different variant; the red line in the middle shows 50% heterozygosity. Note that for most regions, there is no difference between the SI and SC pools, whereas on the short arm of chromosome 5 (and near the centromere) and the long arm of chromosome 7 there are extended regions where the SC pool is more homozygous than the SI pool and skewed towards values near 0 (indicating that it is the same as the AL4 sequence); several examples are shown with arrows on the two chromosomes. The most concentrated region showing this pattern is between 9 and 10 Mb on chromosome 7, the location of the *S*-locus.

Although patterns of polymorphism in each of the pools were very similar (SHOREmap output, Supplementary Figure S5), there were extended regions on both chromosomes 5 and 7 where the SC pool appeared to have low heterozygosity and to be similar to the AL4 reference sequence (that is, dipped towards the 0 side of the graph; Figure 2), whereas the SI pool was polymorphic. The largest region of extended homozygosity was observed between 9 and 10 Mb on the long arm of chromosome 7, the location of the *S*-locus in *A. lyrata* (Hu *et al.*, 2011).

Inspection of SNP calls across the *S*-locus region revealed that although variants were clearly present in the flanking genes and in a fragment of *SCR*, no variants were called at the *SRK* gene (Table 3). As we know this gene should be highly polymorphic, we concluded that it was too divergent to be mapped to the reference. Nevertheless, we noted that the entire flanking gene region (starting from genes upstream from B70 and continuing downstream from B160) showed extensive homozygosity in the SC pools (with variants shared with the AL4 sequences) but polymorphism in the SI pools (Table 3). Mapping of the raw reads identified all three parental B80 alleles in the SI pool but only that associated with S_1_ in the SC pool. Direct B80 genotyping of the F_2_ individuals confirmed this pattern.

Outside of the *S*-locus region on chromosome 7, we also found evidence for the predicted patterns of association with the SC phenotype based on SNP calls from SHOREmap: out of 55 660 SNPs on the long arm of chromosome 7 (excluding indels), 67 were fixed in the SC pool (based on a threshold of 0.1% polymorphism) but different or polymorphic in the SI pools and shared with AL4 but not the MN47 reference sequence. This pattern was most concentrated in two regions in close proximity to each other but some distance from the *S*-locus (positions 6 788 674 to 6 788 963 and 7 382 799 to 7 382 948): 15 SNPs in a gene associated with pollen tube development (β-galactosidase) (Rejon *et al.*, 2013) and 16 SNPs in an unidentified protein adjacent to a gene that has been associated with the SI reaction in *Brassica* (P-loop containing nucleoside triphosphate hydrolase superfamily protein) (Wang *et al.*, 2014). Inspection of the consensus sequences generated by piling up the short reads confirmed that the SC pool was homozygous, whereas the SI pool was heterozygous for both of these regions; homologues of both genes are located on chromosome 4 of *A. thaliana* (positions 13 246 742 to 13 245 999 and 12 809 347 to 12 808 981). Although the SHOREmap output suggested that there also might be candidate genes that differed between the SC and SI pools on chromosome 5, we did not find any regions showing a concentration of homozygosity in the SC pool that was shared with AL4 but not with MN47 or the SI pool.

A more targeted sequence-based examination of variation at the *S*-locus confirmed that the *S*-locus was homozygous in the SC pool but heterozygous in the SI pool. Although neither *SRK* nor *SCR* were mapped to the consensus sequences, the *S*-locus flanking genes (B70, B80, *ARK*3, B120 and B160) were found to be complete on scaffold 7 (Supplementary Figure S6) and were homozygous in the SC pools but heterozygous in the SI pools. There was no difference between the two pools for any of the unlinked genes: *Aly*7 (scaffold 8); *Aly*9 (scaffold 3); *ARK1* and *ARK2* (scaffold 2); *ARC1* (scaffold 4) and *PUB17* (scaffold 1). All but *ARC1* were heterozygous in both pools; *ARC1* showed no nonsynonymous mutations compared with the published functional allele (which was from the MN47 reference). Although the unlinked 13-2 allele was known to be present in both parents, it did not map to the consensus pools, likely because this locus is absent from the MN47 reference sequence.

Mapping the unassembled reads to the expected *SRK* alleles in the crosses (S_1_ and S_23_) clearly confirmed that *SRK* was missing from the SHOREmap SNP calls because it had not been mapped to the reference. The unlinked 13-2 sequence was present in the unmapped reads of both the SI and SC pools but none of the other flanking genes were. For *SRK*, both S_1_ and S_23_ alleles were present in the SI pool but only S_1_ was identified in the SC pool. Mapping the unassembled reads to the full-length *SCR* and *SRK* sequences obtained from the BAC clone for S_1_ (Goubet *et al.*, 2012) demonstrated that both pools had complete sequences for both recognition genes for this haplotype. Moreover, although there were multiple synonymous SNPs compared with the BAC sequences, there were no nonsynonymous mutations that would indicate disruption of function at either gene.

## Discussion

Our results demonstrate that the shift to inbreeding in North American Great Lakes populations of *A. lyrata* is associated with a reduction in the number of SI haplotypes, consistent with theoretical predictions and other experimental studies (reviewed by Vekemans *et al.*, 2014). Based on *SRK* and its flanking genes, we conclude that two *S*-locus haplotypes are associated with this transition across multiple genetic backgrounds but that these are also found in SI individuals from outcrossing populations, potentially reflecting the very young age of the loss of SI. The bulked segregant analysis of F_2_ progeny resulting from a cross between SI and SC parents indicated that loss of SI (at least in the genetic background tested) is recessive and may be associated with a modifier of expression of the S_1_ recognition genes or downstream components of the SI reaction, rather than mutations at the *S*-locus itself. Although further experiments are required to unravel the specific mechanisms, combining new technologies with classical genetic approaches has revealed new insights into a long-standing question.

### Characterisation of *S*-haplotypes in inbreeding and outcrossing populations

The multipronged approach to *S*-locus genotyping used in this study revealed a much clearer pattern of association between *S*-haplotypes and inbreeding than observed in our previous study (Mable and Adam, 2007). The MiSeq pilot study holds promise for more effective utilisation of short read sequencing approaches as an alternative to previous amplicon-based approaches (for example, using 454 sequencing; Jørgensen *et al.,* 2012). We would recommend using the *de novo* sequencing approach for identifying variants, as assembling to a known database was more error prone. Moreover, the tight association between B80 and *SRK* variants confirmed in our study provides a useful tool for resolving genotypes with direct Sanger sequencing.

Based on our new genotyping, one haplotype (S_19_) that has been found to be dominant based on segregation analyses (Prigoda *et al.*, 2005) was overrepresented in the inbreeding populations but underrepresented in the outcrossing populations. Its flanking genes suggest a common origin of S_19_ in the inbreeding populations, as the associated alleles in the flanking genes are monophyletic and show little variation overall. The same variants are found in some individuals from outcrossing populations but these remain strongly SI; we also did not observe homozygotes in the outcrossing populations that would suggest disruption of S_19_. One of the inbreeding populations (LPT) has a single bp mutation in both the *S*-domain (the recognition domain) of *SRK* and in B80 compared with other populations (and also has a unique variant of B120) but this *S*-locus haplotype is not found in the other inbreeding populations or in the outcrossing populations. These results could be explained by: (1) disruption of S_19_ only in the inbreeding populations (for example, by mutations in *SCR* or recombination between *SRK* and *SCR*); (2) presence of a modifier specific to SC individuals suppressing the expression of S_19_; or (3) S_19_ in the inbreeding populations having risen to high frequency because of colonisation history rather than a causal relationship with the breakdown of SI. Enforced selfing of SI individuals with S_19_ could be used to determine whether homozygosity of this haplotype is sufficient for disruption of SI or whether other factors found only in SC individuals are required.

The other main *S*-haplotype occurring in inbreeding populations (S_1_) was found in all populations surveyed except for the inbreeding populations KTT, LPT and TC. This *SRK* allele has been found worldwide, is completely recessive to all other alleles tested and is shared with other Brassicaceae relatives (Schierup *et al.*, 2001; Mable *et al.*, 2004; Paetsch *et al.*, 2006; Castric *et al.*, 2010) but it has not previously been directly associated with loss of SI. We predicted that of the 123 individuals with S_1_ in the Great Lakes populations, 47% were S_1_S_1_ homozygotes. However, 20% of these were found in outcrossing populations, suggesting that it is not just homozygosity for S_1_ that causes loss of SI. In fact, only six fully SC individuals were found in outcrossing populations; although they all contained S_1_, four of these were predicted to be heterozygotes (three with S_13_ and one with an unidentified haplotype; Supplementary Table S4). Although S_1_ was associated with at least 18 distinctive B80 haplotypes (Figure 1), only 2 were found in inbreeding populations. Similarly, only three of the wide range of S_1_ B120 haplotypes were found in the inbreeding populations (Supplementary Figure S1). This is consistent with a bottleneck in *S*-haplotypes, as has been suggested for all other species compared so far (Vekemans *et al.*, 2014).

### Number of *S*-alleles in outcrossing populations

Also striking from our analyses is the relatively low number of *S*-haplotypes predicted in most of the North American populations, including those that are highly outcrossing. This is surprising given that it has been estimated that there should be >100 *S*-haplotypes in outcrossing species (Castric and Vekemans, 2004) and previous surveys of European *A. lyrata* have predicted 16–25 haplotypes per population (Mable *et al.*, 2003; Schierup *et al.*, 2008). The lower number of *S*-haplotypes in the Great Lakes populations is consistent with a predicted bottleneck in North American compared with European populations (Ross-Ibarra *et al.*, 2008). The outcrossing populations with the lowest predicted numbers of *S*-alleles (HDC and OWB) also had relatively low outcrossing rates (*T*_m_=0.65 and 0.64, respectively), more SC and leaky SI than SI individuals and high S_1_ frequency (Supplementary Table S4). These populations thus could be in a transition to inbreeding, possibly because of mate limitation, which has been suggested as a primary driver of transitions from outcrossing to inbreeding (Byers and Meagher, 1992; Vekemans *et al.*, 1998, 2014). If mate limitation occurred during a colonisation bottleneck (that is, after the last ice age), high levels of biparental inbreeding could have purged deleterious mutations that maintain outcrossing in other parts of the range of *A. lyrata* (Sletvold *et al.*, 2013).

### Genetic basis of loss of SI

Our crossing data demonstrated that all F_1_ offspring from the SI × SC cross were strongly SI but SC segregated in the F_2_ generation, indicating that SC as a phenotypic state was recessive. The appearance of male sterile plants (for which the selfing phenotype cannot be determined) made it difficult to estimate the exact proportion of SC plants in the F_2_. Formal testing of genetic models was therefore not possible (Supplementary Table S9). Nevertheless, the fact that all SC plants were homozygous for S_1_, combined with the fixation of S_1_ in the predominantly selfing RON and PTP populations, strongly suggests that SC is functionally linked to this *S*-allele. This association with S_1_ does not appear to be explained by genetic linkage, because S_1_ homozygosity alone was not sufficient to confer SC. One plant in the F_2_ progeny (Supplementary Table S3) and 19 plants raised from wild-collected seeds were S_1_S_1_ but SI (Supplementary Table S4). S_1_ homozygotes that are not SC can be explained by a model that invokes a recessive modifier unlinked to the *S*-locus (scenario A in Supplementary Table S9). Segregation distortion at the *S*-locus is not unprecedented (Bechsgaard *et al.*, 2004). Still, the observation that there was a bias against S_1_ in only half of the crosses among F_1_ plants generated from the same parents (Supplementary Table S8) is also consistent with an unlinked but allele-specific viability modifier, which could explain the low numbers of SC plants surviving to flowering. We conclude that an unlinked, recessive modifier of the expression of S_1_ or its associated downstream genes confers SC in S_1_ homozygotes, at least in the RON and PTP populations.

Results from crosses involving PTP and MAN (Supplementary Figure S4) are similarly consistent with a modifier segregating in outcrossing populations that is only expressed in S_1_ homozygotes that are also homozygous for the modifier. First, even crosses between SI plants can result in SC offspring. Second, crosses between SI individuals from an outcrossing population (MAN, population S_1_ frequency 7/8, Supplementary Table S4) and SC individuals from an inbreeding population (PTP, population fixed for S_1_) yielded variable ratios of SC progeny, generally well below 50%. Although complete *SRK* genotypes were not resolved for the crosses, SC individuals were only found in cases where the SI parent had S_1_ (MAN18b, MAN22f but not MAN17f; Supplementary Figure S4). In the MAN population survey, two individuals were S_1_S_1_ homozygotes but only one was phenotypically SI, even though they shared the same B80 and B120 genotypes (Supplementary Table S4). Further work is needed to identify the exact locus of the recessive modifier of S_1_ or its associated downstream genes, its exact functioning and to test whether it is the only mechanism for the loss of SI in *Arabidopsis lyrata*.

The SHOREmap analysis helped us to predict genomic regions where there was a difference between SI and SC individuals that could be used to initiate this search for the potential modifiers, despite previous concerns that it would only be useful for very large sample sizes (Austin *et al.*, 2011). The bulked segregant analysis indicated that the two pools differed predominantly on chromosomes 5 and 7, with the largest region of homozygosity at the *S*-locus, between 9 and 10 Mb on the long arm of chromosome 7 (Supplementary Figure S5). However, relying only on the SNP-based analysis would have provided misleading results because *SRK* reads could not be aligned to the reference owing to the high divergence between the S_13_ haplotype found in the MN47 reference and the S_1_ and S_23_ haplotypes expected in the pools. It is thus critical for such highly polymorphic genes to include unassembled reads in comparative analyses.

For both the male and female SI recognition genes (*SCR* and *SRK*) and all of the flanking genes tested, comparison of the consensus sequence alignments confirmed that the SC pools were homozygous for S_1_-associated variants, whereas the SI pools were polymorphic. This was in contrast to other members of the *SRK* gene family located on other chromosomes, for which there was no difference in heterozygosity between the pools. There were also no differences detected between the pools in genes that have been implicated in the downstream regulation of SI (*ARC1* and *PUB17*) (Indriolo *et al.*, 2012; Goring *et al.*, 2014; Nasrallah and Nasrallah, 2014). *ARC*1 was homozygous in both pools, and although there were some regions where the pools did not align to the published sequence, these were the same in the SI and SC pools. The role of *PUB17* in the SI reaction is less clear (Goring *et al.*, 2014; Nasrallah and Nasrallah, 2014) but it was heterozygous in both pools. The S_1_ BAC clone was obtained from an individual from Iceland (Goubet *et al.*, 2012), and hence some variation would be expected within this recessive specificity (Castric *et al.*, 2010). However, none of the mutations resulted in amino acid substitutions or altered the reading frame to cause a premature stop codon in *SRK* or *SCR*. We thus have no evidence that mutation of the recognition genes themselves has disrupted SI in this cross.

The analysis of SNPs that were homozygous in the SC pool, shared with the AL4 reference and heterozygous or different in the SI pool, identified two candidate genes for unlinked modifiers. Both genes have previously been implicated in processes that could affect the SI reaction (that is, prevention of pollen tube development). The P-loop containing nucleoside triphosphate hydrolase superfamily protein has been predicted to influence indirect interactions between a network of SI-related genes (identified by comparing SI and SC Chinese cabbage) related to energy metabolism and stress responses in *Arabidopsis* (Wang *et al.*, 2014). In lilies, β-galactosidase has been hypothesised to contribute to degradation of large polysaccharides secreted by papillae in order to allow them to be incorporated into the growing pollen tube (Rejon *et al.*, 2013). Thus, loss of SI could be associated with regulatory changes that affect the complex network of pathways that normally prevent self-pollen from maturing and producing full-length pollen tubes. Additional experiments to investigate the role that such enzymes play in self- and non-self-pollen reactions in *Arabidopsis* species would be necessary to confirm their direct involvement, but the bulked segregant analysis even of small pools of 10 individuals provided a useful starting point for narrowing down the search.

## Conclusions

Our results suggest that there has been a bottleneck in *S*-alleles associated with a transition to inbreeding in *A. lyrata* but that the mechanisms for loss of SI might be because of modifiers of the SI reaction rather than mutations at the *S*-locus itself. They thus emphasise the importance of considering the two processes separately. The situation found in *A. lyrata* where SC segregates in all populations but only rises to high frequency in some provides excellent opportunities for resolving the specific mechanisms and selective forces that promote transitions in mating system. Nevertheless, even in this young system, uncovering the genetic basis of this complex transition remains challenging.

Resolving the chicken-and-egg story of relating current differences between inbreeding and outcrossing populations to the original cause of loss of SI has been of interest to the Population Genetics group community for many years. It thus seems fitting for this fiftieth anniversary to use this type of case study to highlight the benefits of continuing to embrace the vast contributions that have been made by ‘old school' classical genetics when implementing new technologies to answer old questions. It is the combination of approaches that has most power to reveal new insights.

## Data archiving

Data for this study (SNP calls for chromosomes 5 and 7 from the SC and SI pools, alignments of B70, B80, B120 and B160 haplotypes) are available from the Dryad Digital Repository: http://dx.doi.org/10.5061/dryad.832t8. Short-read data from the MiSeq amplicon sequencing and the Illumina bulked segregant analysis of SC and SI pools are available from the National Centre for Biotechnology Information BioProject ID: PRJNA339675. Sanger sequencing data are available from GenBank, with the following accession numbers: KX923797 for the new AlySRK52 sequence; KX923776-KX923796 for B70, and KX923711-KX923775 for B120.

## Figures and Tables

**Table 1:** Number of *SRK* alleles inferred for 8 individuals per population based on direct and indirect genotyping, sorted by genetic cluster predicted by STRUCTURE analysis of microsatellites and population outcrossing rate (both taken from Foxe *et al.*, 2010) indicating the number of individuals containing each of the variants screened in the allele-specific genotyping, as well as other alleles identified by cloning or MiSeq analyses

*Population*	*T_m_*	*Cluster*	*SRK alleles inferred*							*Other*	*% Het*	*Homo*	*Min alleles*^a^	*Max alleles*^a^
			*S*_*1*_	*S*_*3*_	*S*_*13*_	*S*_*19*_	*S*_*20*_	*S*_*23*_	*S*_*39*_					
TC	0.18	1	0	1	0	8	0	0	0		0.14	S_19_	2	2
TCA	0.48^c^	1	0	0	0	8	0	0	0		0.00	S_19_	1	1
TSSA	0.41	1	4	1	3	4	0	0	0	S_27_	0.63	S_1_, S_19_	6	6
TSS	0.91	1	3	4	3	2	0	0	0	S_27_	0.88	S_3_	6	11
PTP	0.02	2	8	0	0	0	0	0	0		0.00	S_1_	1	1
WAS	0.25	2	4	0	0	8	0	0	0		0.50	S_19_	2	2
RON	0.28	2	8	0	0	0	0	0	0		0.00	S_1_	1	1
PIN	0.84	2	7	1	0	3	1	0	4		1.00	None	6	6
PCR	0.98	2	8	0	0	0	1	2	0	S_27_, S45^d^	0.75	S_1_	5	8
KTT	0.31	3	0	0	0	8	0	0	0		0.00	S_19_	1	1
PIR	0.88	3	7	2	0	0	0	1	2		0.75	S_1_	6	14
LPT	0.13	4	0	0	0	8	0	0	0		0.13	S_19_	2	2
HDC	0.65	4	8	0	0	0	0	0	0	S45^d^	0.38	S_1_	3	3
PRI	0.89	4	8	0	0	1	0	0	1		0.75	S_1_	5	8
OWB	0.64	5	8	0	1	0	0	0	0		0.13	S_1_	2	2
PIC	0.77	5	8	2	0	0	0	0	0	S45^d^	0.50	S_1_	3	6
LSP	0.94	5	8	0	0	0	0	2	1		0.75	S_1_	3	8
SBD	0.94	5	5	4	1	1	1	0	0	S45^d^	0.88	S_1_	8	14
PUK	0.96	5	2	5	0	5	0	1	2		0.88	S_3_	6	6
BEI	0.98	5	7	2	1	1	0	0	0		0.50	S_1_	5	5
IOM	0.94	6	4	4	0	0	0	0	0		0.63	S_1_	3	14
NCM	0.99	6	6	6	1	0	0	0	1	S45^d^	0.75	S_1_, S_3_	5	5
MAN	0.83	1, 2	7	0	0	0	1	0	1	S_27_, S52^e^	0.75	S_1_	8	11
IND	0.98	2, 5	4	4	1	0	1	0	1	S45^d^	0.88	S_1_	5	14
**Inbreeding**	**0.20**		**0.36**	**0.02**	**0.00**	**0.71**	**0.00**	**0.00**	**0.00**		**0.11**		**1.43**	**1.43**
**Outcrossing**^b^	**0.88**		**0.79**	**0.27**	**0.06**	**0.10**	**0.04**	**0.05**	**0.10**		**0.70**		**5.13**	**8.63**

Abbreviations: Het, heterozygous; Homo, homozygous; Min, minimum; Max, maximum; SRK, *S*-locus receptor kinase.

For each population, the % of individuals predicted to be heterozygous, the alleles predicted to be homozygous and the minimum and maximum number of alleles in the population predicted by the repeatability index of Stevens and Kay (1989) are shown. Rows in bold indicate the average outcrossing rates, proportion of individuals with each *SRK* allele, heterozygosity and maximum and minimum number of alleles predicted for inbreeding and outcrossing populations. See Supplementary Table S7 for full details.

a Minimum number of alleles calculated assuming all missing alleles in a population were the same; maximum assuming all were different.

b Calculated excluding the mixed mating population TSSA.

c Outcrossing rate was based on only 5 families and 5 individuals per family, and hence it was excluded from calculation of averages.

d S45 is unlinked to the *S*-phenotype and sometimes found with two other *SRK* alleles but only present in some individuals.

e A putatively new allele was allocated the name S52 but phenotypic testing of linkage would be required before official naming as an *S*-allele.

**Table 2:** Flanking gene variants for individuals that showed amplification of only one *SRK* allele (using direct allele-specific screening) and were homozygous at B80

*SRK*	*B80*	*B120*	*B160*	*B70*^a^	*Phenotype*	*Population*^b^	N
19	75	25	15	1	SC	KTT	6
19	75	25	15	?	SC	KTT	2
19	75	59	15	1	SC	TCA	1
19	75	?	15	1	SC	TCA	1
19	75	59	15	?	SC	TCA	1
19	75	6	15	?	SC, SI	TC	5
19	75	24	15	?	SC	TC, TCA	4
19	75	?	15	?	SC	TC, TCA	5
19	75	24	15	?	SC	*TSSA*	2
19	75	40	15, 45	?	SC	WAS	1
19	75	40	22	?	SC	WAS	1
19	75	64	15	?	SC	WAS	2
19	75	64	15, 16	?	SC	WAS	1
19^c^	76	26	22	?	SC	LPT	7
1	50	7	16	2	PC	**HDC**	1
1	50	7	16	?	PC, SI	**HDC**	2
1	50	7	21	2	PC	**HDC**	1
1	50	7,8	19, 20	2	PC	**HDC**	1
1	50	7	22	17	SC	RON	3
1	50	7	22	?	SC	RON, PTP	11
1	50	7	23	?	SC	RON	1
1	50	?	22	?	SC	RON	1
1	51	47	38	14	SI	**PRI**	1
1	67	20	16	4	PC	**IOM**	1
1	67	20	16, 24	?	SI	**IOM**	1
1	78	3	15	11, 12	SI	**LSP**	1
1	78	3	15, 22	?	SI	**LSP**	1
1	115	3	15, 41	4	SI	**SBD**	1
1	43	?	15	?	SI	*TSSA*	1
3	48	6	15	20	SI	**TSS**	1
3	48	?	40	15	SI	**PUK**	1

Abbreviations: PC, partially self-compatible; SC, self-compatible; SI, self-incompatible; SRK *S*-locus receptor kinase.

Numbers indicate the allele designation at *SRK* and its flanking genes; unresolved alleles are indicated by ‘*?*'. For each *S*-locus haplotype (that is, combination of alleles), the selfing phenotype, population and the number of individuals (*N*) in which it was found are indicated.

a B70 showed unreliable amplification but some genotypes were resolved.

b Outcrossing populations are in bold; the mixed mating population *TSSA* is in italics.

c Single synonymous mutation in *S*-domain of *SRK* compared with other populations.

**Table 3:** SNP analysis of the *S*-locus region (9–10 Mb on chromosome 7) from the SHORE output, indicating the identity of the gene, the number of SNPs called, the number of SNPs in coding regions and the proportion of sites for each gene that: (1) were homozygous in the SC pool; (2) homozygous in both the SC pool and AL4 sequences but not in the SI pool; (3) were fixed for different variants in the SI and SC pools when both were homozygous; (4) shared the same SNP in the SC pool and the AL4 sequences when both were homozygous but the SI pool was heterozygous; (5) showed the same homozygous variant in the SC pool as found in the MN47 reference; (6) showed incomplete coverage in the SC pool, indicated by missing variants because of lack of reads; and (7) had indels in regions that did show read coverage

*Gene*^a^	N *SNPs*	N *SNPs coding*	*(1) Homo SC*	*(2) AL4 and SC both Homo but SI Het*	*(3) SC≠SI when both Homo*	*(4) SC=AL4 when Homo and SI Het*	*(5) SC=MN47 when SC Homo*	*(6) Sites missing in SC*	*(7) Indels*
**T6K22.160 (B160, transcription factor)**	25	14	0.88	0.20	0.20	0.20	0.20	0.20	0.12
T6K22.150	4	4	1.00	0.50	0.00	0.50	0.50	0.00	0.00
T6K22.140	19	13	0.47	0.05	0.00	0.05	0.05	0.00	0.00
T6K22.130	145	105	0.96	0.54	0.14	0.45	0.46	0.09	0.03
**T6K22.120 (B120, lectin protein kinase)**	75	64	1.00	0.49	0.00	0.45	0.45	0.00	0.05
**T6K22.110 (ARK3)**	207	137	0.88	0.41	0.18	0.34	0.34	0.07	0.12
**T6K22.90 (SCRA**)	38	0	0.32	0.16	0.11	0.13	0.18	0.13	0.08
**T6K22.100 (SRK)**	**0**								
**T6K22.80 (B80, PUB8)**	38	36	1.00	0.53	0.53	0.53	0.53	0.00	0.00
**T6K22.70 (B70, ethylene responsive)**	9	5	0.78	0.22	0.00	0.22	0.22	0.00	0.11
T6K22.60 (TF dysfunctional tapetum)	28	11	1.00	0.96	0.96	0.96	0.96	0.00	0.14
T6K22.50 (subtilase family protein)	91	53	0.97	0.38	0.00	0.38	0.38	0.00	0.02
T6K22.40 (unknown protein)	34	15	1.00	0.56	0.00	0.56	0.56	0.00	0.03
T6K22.30 (pentatricopeptide repeat-containing protein)	59	59	1.00	0.32	0.00	0.32	0.32	0.00	0.00
T6K22.20 (oxygen-evolving enhancer protein 3-1)	27	1	0.44	0.11	0.00	0.11	0.11	0.00	0.89
T6K22.10 (KatA mRNA for kinesin-like motor protein)	17	1	1.00	0.47	0.00	0.47	0.47	0.00	0.24

Abbreviations: Het, heterozygous; Homo, homozygous; SC, self-compatible; SI, self-incompatible; SNP, single-nucleotide polymorphism; SRK, S-locus receptor kinase; TF, transcription factor.

Flanking genes screened in the population survey are indicated in bold. Note that no SNPs were called in the region where *SRK* was anticipated to be located.

a T6k22 numbers are from the bacterial artificial chromosome (BAC) clone sequenced by Kusaba *et al.* (2001).
