# What causes mating system shifts in plants? Arabidopsis lyrata as a case study

**DOI:** 10.1038/hdy.2016.122

**Published:** 2016-12-07

**Authors:** B K Mable, J Hagmann, S-T Kim, A Adam, E Kilbride, D Weigel, M Stift

**Correction to:** *Heredity* (2016) **118**, 52–63; doi:10.1038/hdy.2016.99; published online 2 November 2016

**Updated online 7 December 2016:** This article was originally published under NPG's License to Publish, but has now been made available under a CC BY 4.0 license. The PDF and HTML versions of the paper have been modified accordingly.

The corrected article appears in this issue together with this corrigendum. The publisher wishes to apologize for any inconvenience caused.

