# Supplementary material for: What causes mating system shifts in plants? Arabidopsis lyrata as a case study: Updated online 7 December 2016: This article was originally published under a standard licence, but has now been made available under a CC BY 4.0 licence. The PDF and HTML versions of the paper have been modified accordingly. A corrigendum has also been published
Source: Heredity (Edinb). 2016 Nov 2;118(1):52–63. doi: 10.1038/hdy.2016.99 (PMC5176122; doi:10.1038/hdy.2016.99)
Supplement: Supplementary Methods [file hdy201699x1.docx]

**Supplementary Methods**

***Cloning and Tagged Amplicon Sequencing Protocols***

For traditional cloning (using Invitrogen TA cloning kits for sequencing; Invitrogen Inc., Paisley), we targeted a subset of individuals (1-6 individuals per outcrossing population, using degenerate primers (13FBM and SLGR), as described in Mable *et al.* (2004) (Supplementary Table S2). We then sequenced up to 18 clones per individual per primer set. Resulting amplicons were dominated by members of the gene family not physically linked to the *S*-locus (particularly *Aly*9 and *ARK*3). We thus also tested two other primer combinations involving a degenerate forward primer with SLGR: an unpublished primer (13-3sF) that was developed for the study described by Charlesworth *et al*.(2003) and a newly designed degenerate primer that was designed to target a conserved region at bp 497 of the *SRK* gene (SRK497F), based on an alignment of all available published sequences, along with additional unpublished sequences from our laboratory whose linkage to the SI phenotype has not been established, again sequencing up to 18 clones per individual per primer set.

The primers 13-3sF and SRK497F were also used to pilot a technique to allow sequencing of 900 bp amplicons using short read technologies, since there are no conserved regions within the 500 bp limit required by standard tagged amplicon approaches. For this pilot study, PCR was performed on DNA extracted from three individuals per population from seven outcrossing populations (HDC, IND, MAN, PCR, PIN, SBD, TSS) and the mixed mating population (TSSA), using the two degenerate forward primers (13-3sf; SRK497: Supplementary Table S2) and a barcoded version of the common reverse primer (SLGR). Amplicons were gel purified and quantified using a Qubit fluorometer, equal amounts of PCR products pooled for each individual, and sent to the Centre for Genome Research at the University of Liverpool for sequencing on a Miseq (Illumina).

From each amplicon, 731 to 1092 ng was sheared using the Covaris S2 sonicator and used as input material for the TruSeq DNA LT Sample Prep Kit (Illumina). Following 6 cycles of amplification, using the KAPA HiFi HS ReadyMix, libraries were purified using AMPure XP beads. Each library was quantified using Qubit and the size distribution assessed using the Agilent 2100 Bioanalyser. These final libraries were pooled in equimolar amounts using the Qubit and Bioanalyzer data. The pool was size-selected at 520-920 bp using the Sage Pippin Prep.The quantity and quality of the pool was assessed using the Qubit Bioanalyzer , followed by qPCR using the Illumina Library Quantification Kit from Kapa on a Roche Light Cycler LC480II, according to manufacturer's instructions. The pool of libraries was sequenced on one flowcell of the MiSeq at 2x250 bp using paired-end sequencing chemistry.

Raw reads were separated by barcode and assembled into individual FASTQ files for singlets (R0), forward (R1) and reverse (R2) reads. The raw Fastq files were trimmed for the presence of Illumina adapter sequences using [Cutadapt](http://code.google.com/p/cutadapt) version 1.2.1 (Martin, 2011). The option -O 3 was used, so the 3' end of any reads which match the adapter sequence for 3 bp. or more are trimmed. The reads were further trimmed using [Sickle](https://github.com/najoshi/sickle) version 1.200 (Joshi NA and Fass JN. 2011. Sickle: A sliding-window, adaptive, quality-based trimming tool for FastQ files. Available at <https://github.com/najoshi/sickle>) with a minimum window quality score of 20. Reads shorter than 10 bp. after trimming were removed. If only one of a read pair passed this filter, it was included in the R0 file. The median target trimmed read length was 250 bp. Summary statistics were generated using the fastq-stats from EAUtils (Erik Aronesty. 2011. ea-utils : "Command-line tools for processing biological sequencing data"; <http://code.google.com/p/ea-utils>).

Trimmed paired reads (i.e. the R1 and R2 files combined) from each individual initially were aligned to a database of all known *SRK* alleles and its broader gene family (see (Jorgensen *et al*, 2011) using the “assemble to reference” protocol implemented in CLC genomics workbench (version 7.5, Qiagen Aarhus). Consensus sequences were extracted from all matches to the database to confirm their identity using Mega BLAST, as implemented through the National Centre for Biotechnology Information (NCBI) website. In cases where sequences were heterozygous, an ambiguity threshold of 0.2 and minimum of 20 reads was set to add IUPAC ambiguity codes to generate consensus sequences. In addition to the predictions based on B80 genotyping, another member of the *SRK* gene family that was identified in all individuals by the MiSeq analysis (*Aly*8 or *Ark*3), was also checked to see whether it could be used to predict when alleles had been missed for each individual. This is known to be one of the genes most tightly physically linked to *SRK* (Roux et al. 2013) and shows high polymorphism.

In an attempt to resolve more alleles and to resolve ambiguities due to alignment of short reads to multiple reference sequences, an alternative approach was also used. It is the results of this analysis that are reported in the main text. The trimmed pair-end reads were first assembled *de novo* using CLC genomics, setting a minimum contig length of 450, and using default parameters of automatic word and bubble size. Raw reads were then mapped back onto the contigs using a mismatch and insertion cost of 3, length fraction 0.8, similarity fraction 0.8 and a global alignment to consider gaps as mismatches. This resulted in reconstruction of full contigs in the target range of 800-940 bp (because different *SRK* alleles and other members of the gene family show extensive length polymorphism), as well as some longer and some shorter contigs. Contigs for each individual were sorted by read number and consensus sequences were extracted. Mega BLAST was then used to identify matches to sequences deposited to GenBank. It was not possible to set a single threshold of contig length prior to screening but no contigs showing fewer than 300 overall reads and less than 100 average reads per site showed significant matches in BLAST. This still resulted in 16-18 different sequence types per individual that were checked using BLAST. In cases where the assemble-to-reference approach had identified an allele not deposited to Genbank, “align two sequences” was used in BLAST to confirm identity of the *de novo* contigs to other alleles in the database. Sequences with at least 80% match to *SRK*-like sequences were identified as putatively new *SRK* alleles. However, confirmation of this would require testing using segregation analyses.

*Bulked Segregant Analysis*

To produce high-yield DNA extractions for Illumina sequencing of separate pools of SC and SI individuals from the F2 crosses, we used a combined approach. Dried leaves were homogenized dry for 30 s at a speed of 4.0 (4 m/s) using a FastPrep FP120 Homogenizer (Thermo Savant Carlsbad, California, USA) with lysing matrix beads (MP Biomedicals, Santa Ana, California, USA). Nuclei were extracted using 0.4 ml cold nuclei extraction buffer (10 mM Tris-HCl 9.5, 10 mM EDTA pH 8.0, 100 mM KCl, 500 mM sucrose, 4 mM spermidine, 1 mM spermine, 0.1% beta-mercaptoethanol). DNA was purified using the Qiagen DNeasy Plant Tissue Kit, mini protocol (Qiagen Ltd, Paisley, UK) and eluted in nuclease-free water. A NanoDrop ND-1000 spectrophotometer (Thermo Scientific, Wilmington, Delaware, USA) was used to measure the concentration and quality of the extracted DNA.

In addition to the published reference sequence for *A. lyrata* (MN47 from the outcrossing IND population), we compared the SI and SC pools to an unpublished sequence assembly for individuals from the inbreeding RON population. Two F_1_ siblings from the RON/MN47 hybrids (labelled AL4) were independently sequenced using Illumina GAII (101bp pair-end run) and reads were mapped against the MN47 reference using GenomeMapper (Schneeberger *et al*, 2009). These two siblings are from part of a whole genome resequencing project (unpublished data); reads uniquely mapped (mean depth: 46-fold) covered around 70% of the reference genome (73.21 and 73.49%, respectively), resulting in 632,059 and 587,674 SNPs for each. The hybrid AL4 genome was then inferred by subtracting from the MN47 sequence.

**Supplementary Results**

***Cloning and Tagged Amplicon Sequencing***

All three sets of degenerate primers showed a bias towards amplification of known members of the SRK gene family that are not involved in the SI response (particularly, *Aly*8 or *ARK*3 and *Aly*9 or *ATS*1; Charlesworth *et al*, 2003) (Supplementary Tables S4 and S5). Although some known *SRK* alleles were also resolved, overall, cloning would not have been useful without screening very large numbers of clones per individual.

For the MiSeq amplicon analysis, all samples met the target of a median of 250 bp median read length but varied in the total number of reads (Supplementary Table S5). The lowest number (0.4 million reads) was found for HDC3 and the highest for MAN6, PCR2, TSSA10 and TSS12 (>1.2 million reads). There was no correlation between the number of reads and the number of *SRK* alleles resolved. In general, the MiSeq analysis resolved more alleles than allele-specific PCR but in all cases, when alleles were amplified using the latter they were also found in the former. In some cases where none of the allele-specific PCR primers tested positive (IND2, IND10, PCR2, PCR5, TSSA8), the MiSeq analysis identified the presence of known *SRK* alleles.

While the initial approach of aligning the trimmed paired-end reads to the reference database of known *SRK*-like gene family members was quicker and easier than the *de novo* analysis, eight individuals that were predicted to be heterozygotes had only one matching allele identified using the former whereas only two remained with unidentified alleles using the latter. Moreover, in the assemble-to-reference analysis it appeared that there was high similarity to a previously described allele in most individuals (Aly*SRK*32; (Tedder *et al*, 2011); however, using the *de novo* analysis, the most similar contig was only 96% identical to the reference database allele, suggesting that it is not actually this allele that is unlinked to the *S*-locus. There were also quite a few instances where there appeared to be high read numbers matching particular reference sequences but BLAST revealed low similarity, when using the align-to-reference approach.

The more thorough approach of *de novo* assembly of the reads and then mapping the reads back onto the resulting contigs appeared to be more accurate, as longer contigs could be assembled for the BLAST analyses and there was less ambiguity (see Supplementary Table S5). For most individuals, 16-18 different contigs were produced at read numbers greater than 300 and an average coverage of at least 100. However, it was not possible to identify *SRK* alleles based on their contig length, read number or average coverage; for example AlySRK3 was identified in a short contig with very low read numbers (Supplementary Table S5). Known members of the *SRK* gene family that are not involved in the SI response amplified in most individuals: *ARK*1 (Aly10.1), *ARK*2 (Aly10.2), *ARK*3 (Aly8), Aly3, Aly9, Aly14, and Aly13-2 (Supplementary Table S5). There were also two other genes that amplified in most individuals that would have been combined as “AlySRK32” using the reference database approach: putative *A. lyrata* proteins XM002868646.1 and XM002870742.1. The MiSeq analysis also identified one new *SRK*-like allele: this allele had assembled to the *SRK*15 reference sequence but was only 80% similar based on the *de novo* contig analysis. One previously identified allele (Aly*SRK*45) was only present in some individuals and some populations but it was sometimes found with two other alleles in the same individual (Supplementary Tables S4 and S5). A segregation analysis had already suggested that it was unlinked to the SI phenotype (Jorgensen et al, unpublished data), emphasizing the importance of combining genetics with the genotyping. The MiSeq approach also requires a good database of known sequences and it remains a challenge to identify putative *SRK* alleles that are not similar to ones that have already been described.

**References**

Charlesworth D, Bartolome C, Schierup MH, Mable BK (2003). Haplotype structure of the stigmatic self-incompatibility gene in natural populations of Arabidopsis lyrata. *Mol Biol Evol* **20**(11)**:** 1741-1753.

Jorgensen MH, Ehrich D, Schmickl R, Koch MA, Brysting AK (2011). Interspecific and interploidal gene flow in Central European Arabidopsis (Brassicaceae). *Bmc Evol Biol* **11:** 346.

Mable BK, Beland J, Di Berardo C (2004). Inheritance and dominance of self-incompatibility alleles in polyploid *Arabidopsis lyrata*. *Heredity* **93:** 476-486.

Martin M (2011). Cutadapt removes adapter sequences from high-throughput sequencing reads. *2011* **17**(1).

Schneeberger K, Hagmann J, Ossowski S, Warthmann N, Gesing S, Kohlbacher O *et al* (2009). Simultaneous alignment of short reads against multiple genomes. *Genome Biol* **10**(9)**:** 1-12.

Tedder A, Ansell SW, Lao X, Vogel JC, Mable BK (2011). Sporophytic self-incompatibility genes and mating system variation in Arabis alpina. *Ann Bot* **108**(4)**:** 699-713.
