# Supplementary material for: What causes mating system shifts in plants? Arabidopsis lyrata as a case study: Updated online 7 December 2016: This article was originally published under a standard licence, but has now been made available under a CC BY 4.0 licence. The PDF and HTML versions of the paper have been modified accordingly. A corrigendum has also been published
Source: Heredity (Edinb). 2016 Nov 2;118(1):52–63. doi: 10.1038/hdy.2016.99 (PMC5176122; doi:10.1038/hdy.2016.99)
Supplement: Supplementary Information [file hdy201699x2.docx]

**Titles and Legends to Supplementary Figures**

**Figure S1:** Minimum evolution genealogy of B120 alleles, indicating associations with *SRK* alleles and geographic distribution. The frequency of each allele is indicated in parentheses after its name. The tree was reconstructed using MEGA 6.0, under a Tamura 3 parameter model of evolution with rate heterogeneity modelled under a gamma distribution with a shape parameter of 0.09. Numbers on the nodes indicate bootstrap support based on 1000 pseudoreplicates. Associated *SRK* alleles are indicated by name and using coloured branches. Occurrences of each B120 allele in inbreeding and outcrossing populations and in each of the six genetic clusters predicted by STRUCTURE are indicated in the table to the right.

**Figure S2:** Minimum evolution genealogy of B160 alleles, indicating associations with *SRK* alleles and geographic distribution. The frequency of each allele is indicated in parentheses after its name. The tree was reconstructed using MEGA 6.0, under a Tamura Nei 93 model of evolution with rate heterogeneity modelled under a gamma distribution with a shape parameter of 0.05. Numbers on the nodes indicate bootstrap support based on 1000 pseudoreplicates. Associated *SRK* alleles are indicated by name and using coloured branches. Occurrences of each B160 allele in inbreeding and outcrossing populations and in each of the six genetic clusters predicted by STRUCTURE are indicated in the table to the right.

**Figure S3:** Minimum evolution genealogy of B70 alleles, indicating associations with *SRK* alleles and geographic distribution. The frequency of each allele is indicated in parentheses after its name. The tree was reconstructed using MEGA 6.0, under a Tamura 3 parameter model of evolution with rate heterogeneity modelled under a gamma distribution with a rate parameter of 0.05. Numbers on the nodes indicate bootstrap support based on 1000 pseudoreplicates. Associated *SRK* alleles are indicated by name and using coloured branches. Occurrences of each B70 allele in inbreeding and outcrossing populations and in each of the six genetic clusters predicted by STRUCTURE are indicated in the table to the right. Note that no B70 alleles were resolved for cluster 6.

**Figure S4:** Distribution of selfing phenotypes in F1 cross progeny. Parents from the highly outcrossing populations MAN and PIN were all self-incompatible (SI); parents from the predominantly selfing populations PTP and RON are from the same population genetic background and were all self-compatible (SC). SRK genotypes are indicated for each parent (S_?_ indicates unidentified alleles). The number of F1 progeny that are: 1) SI is shown in white; 2) predominantly SI but show some leakiness is shown in light grey; and 3) SC is shown in dark grey. The F1 progeny from the PIN x RON cross was used to generate the F2 segregating population used for the bulked segregant analysis. Note that there are variable proportions of SC individuals produced in the MAN x PTP crosses and that although most progeny from the within-population crosses for MAN are SI (or leaky), one SC individual was produced. Moreover, MAN-17f did not carry S_1_ and no progeny were SC. Together, these results support our conclusion that SC in the PTP and RON population is due to the action of a modifier of the expression of S_1_ that confers SC in S_1_ homozygotes. Given the available S-genotypes, and assuming that all SC parents are fixed for the modifier, a genetically unlinked, recessive modifier would explain the observed SC ratios in the MAN x PTP and MAN x MAN crosses better than a dominant modifier unlinked to the S-locus, or a modifier genetically linked to the S-locus.

Crosses involving MAN-22f are particularly informative. Since it is an S_1_ heterozygote, half of the progeny of the crosses with PTP individuals (S_1_ homozygotes) would be S_1_ homozygotes. If the modifier is S-linked, S_1_ homozygotes would by definition express the modifier, so that the predicted SC frequency would be 50% (vs 21% observed, model rejected with P<0.001*). If the modifier is unlinked recessive and MAN-22f is heterozygous for the modifier, only progeny that inherit both S_1_ and the recessive modifier from MAN-22F should be SC, resulting in a predicted SC frequency of 25% (vs 21% observed, P=0.339*). If the modifier is unlinked dominant, all progeny would inherit the modifier from the PTP parent (regardless of whether MAN-22f also carries it), resulting in a predicted SC frequency of 50% (vs 21% observed, model rejected with P<0.001*).

*two-sided exact bionomial test (R)

**Figure S5:** Complete SHOREmap output for all chromosomes. The trace in red shows comparison of the SC pool to the reference sequence AL4 (from an SC individual from RON) and the trace in blue shows that for the SI pool. The scale at the bottom shows the position along the chromosome. The plots were produced using a step size of 10,000 and a window size of 200,000 bp. For each chromosome plot, the Y axis indicates the proportion of reads either matching or showing an alternative to the reference sequence: 0 indicates fixation of variants that match AL4 and 1 indicates fixation for a different variant; the red line in the middle shows 50% heterozygosity. Note that for most regions, there is no difference between the SI and SC pools whereas on the short arm of chromosome 5 (and near the centromere on the long arm) and the long arm of chromosome 7 there are extended regions where the SC pool is more homozygous than the SI pool and skewed towards values near 0 (indicating that it is the same as the AL4 sequence); several examples are shown with arrows on the two chromosomes. The most concentrated region showing this pattern is between Mb 9 and 10 on chromosome 7, which is the location of the *S*-locus.

**Figure S6:** Graph showing mapping of *S*-locus genes to the SC pool consensus pool sequence. B160, B120, ARK3, B80 and B70 were mapped between Mb 9 and 10 on scaffold 7, whereas the recognition genes themselves (*SRK* and *SCR*) did not map to any region in the genome. The plot shows the position on the scaffold along the top, with the coverage of each gene indicated and the length of the consensus sequence that matches.
