# Supplementary material for: What causes mating system shifts in plants? Arabidopsis lyrata as a case study: Updated online 7 December 2016: This article was originally published under a standard licence, but has now been made available under a CC BY 4.0 licence. The PDF and HTML versions of the paper have been modified accordingly. A corrigendum has also been published
Source: Heredity (Edinb). 2016 Nov 2;118(1):52–63. doi: 10.1038/hdy.2016.99 (PMC5176122; doi:10.1038/hdy.2016.99)
Supplement: Supplementary Figures [file hdy201699x3.ppt]

## Slide 1
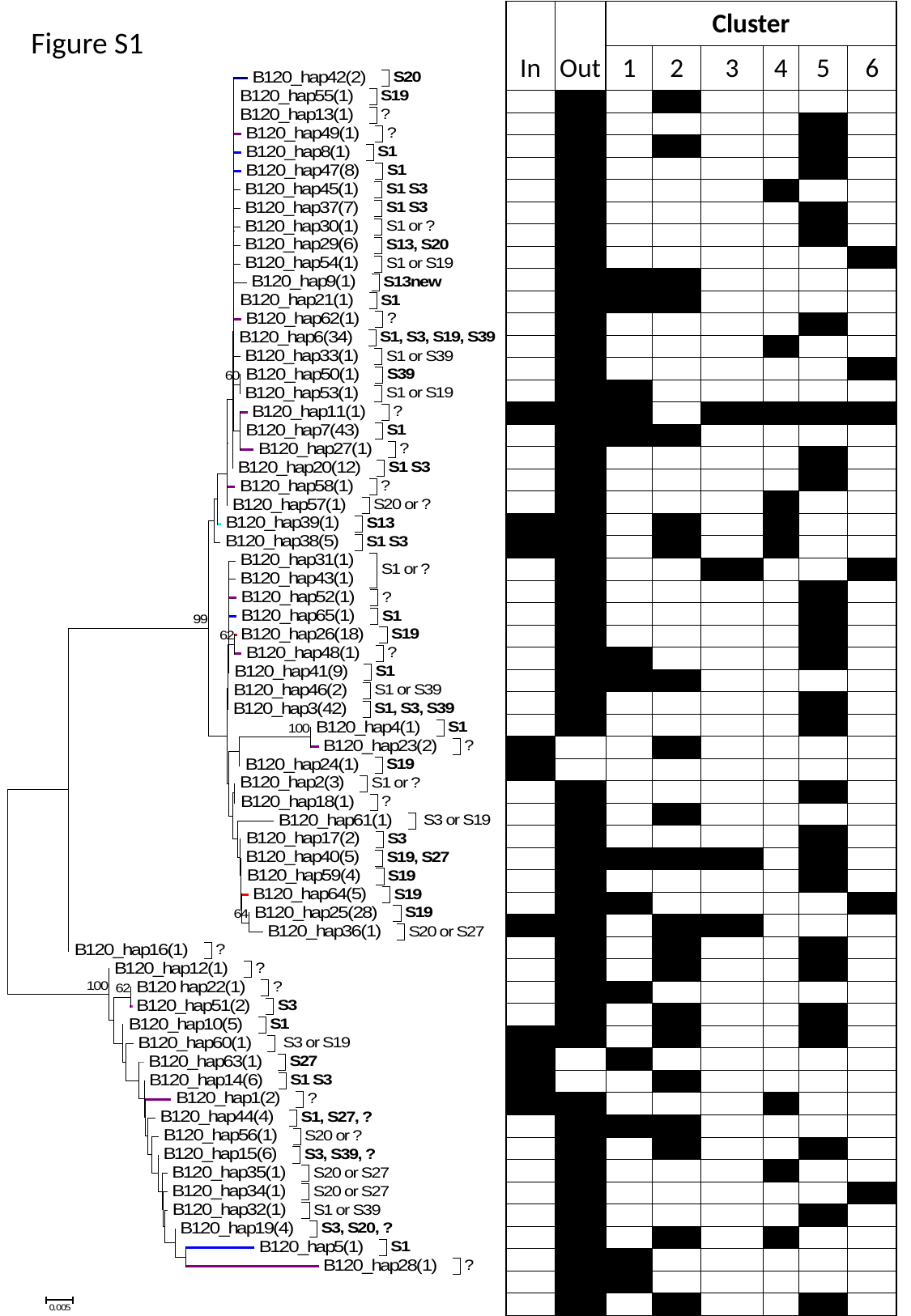

| | | Cluster | | | | | |
| --- | --- | --- | --- | --- | --- | --- | --- |
| In | Out | 1 | 2 | 3 | 4 | 5 | 6 |
| | | | | | | | |
| | | | | | | | |
| | | | | | | | |
| | | | | | | | |
| | | | | | | | |
| | | | | | | | |
| | | | | | | | |
| | | | | | | | |
| | | | | | | | |
| | | | | | | | |
| | | | | | | | |
| | | | | | | | |
| | | | | | | | |
| | | | | | | | |
| | | | | | | | |
| | | | | | | | |
| | | | | | | | |
| | | | | | | | |
| | | | | | | | |
| | | | | | | | |
| | | | | | | | |
| | | | | | | | |
| | | | | | | | |
| | | | | | | | |
| | | | | | | | |
| | | | | | | | |
| | | | | | | | |
| | | | | | | | |
| | | | | | | | |
| | | | | | | | |
| | | | | | | | |
| | | | | | | | |
| | | | | | | | |
| | | | | | | | |
| | | | | | | | |
| | | | | | | | |
| | | | | | | | |
| | | | | | | | |
| | | | | | | | |
| | | | | | | | |
| | | | | | | | |
| | | | | | | | |
| | | | | | | | |
| | | | | | | | |
| | | | | | | | |
| | | | | | | | |
| | | | | | | | |
| | | | | | | | |
| | | | | | | | |
| | | | | | | | |
| | | | | | | | |
| | | | | | | | |
| | | | | | | | |
| | | | | | | | |
| | | | | | | | |
| | | | | | | | |
| | | | | | | | |
| | | | | | | | |
| | | | | | | | |
| | | | | | | | |
| | | | | | | | |
| | | | | | | | |
| | | | | | | | |
| | | | | | | | |
| | | | | | | | |
Figure S1

## Slide 2
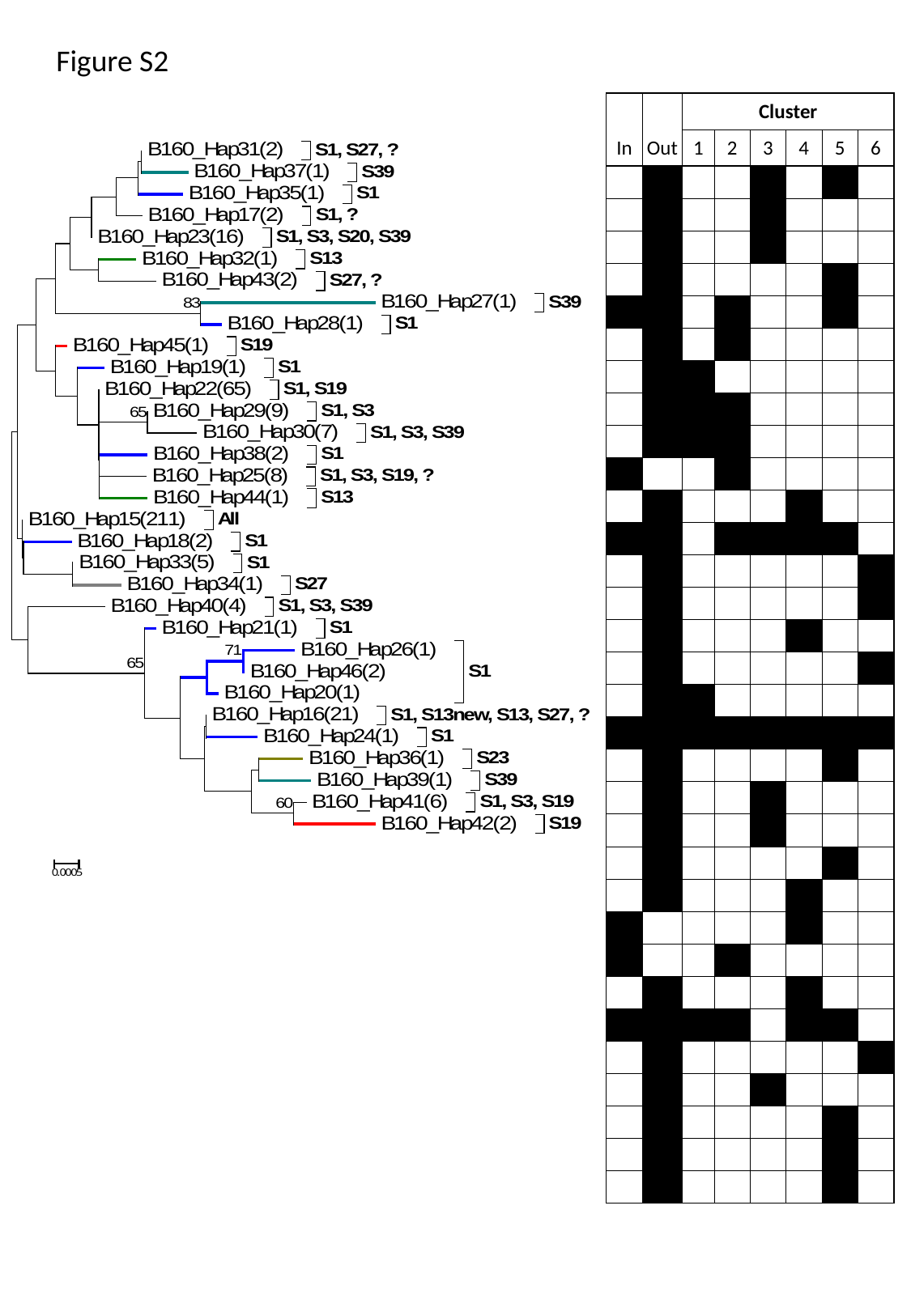

Figure S2
| | | Cluster | | | | | |
| --- | --- | --- | --- | --- | --- | --- | --- |
| In | Out | 1 | 2 | 3 | 4 | 5 | 6 |
| | | | | | | | |
| | | | | | | | |
| | | | | | | | |
| | | | | | | | |
| | | | | | | | |
| | | | | | | | |
| | | | | | | | |
| | | | | | | | |
| | | | | | | | |
| | | | | | | | |
| | | | | | | | |
| | | | | | | | |
| | | | | | | | |
| | | | | | | | |
| | | | | | | | |
| | | | | | | | |
| | | | | | | | |
| | | | | | | | |
| | | | | | | | |
| | | | | | | | |
| | | | | | | | |
| | | | | | | | |
| | | | | | | | |
| | | | | | | | |
| | | | | | | | |
| | | | | | | | |
| | | | | | | | |
| | | | | | | | |
| | | | | | | | |
| | | | | | | | |
| | | | | | | | |
| | | | | | | | |

## Slide 3
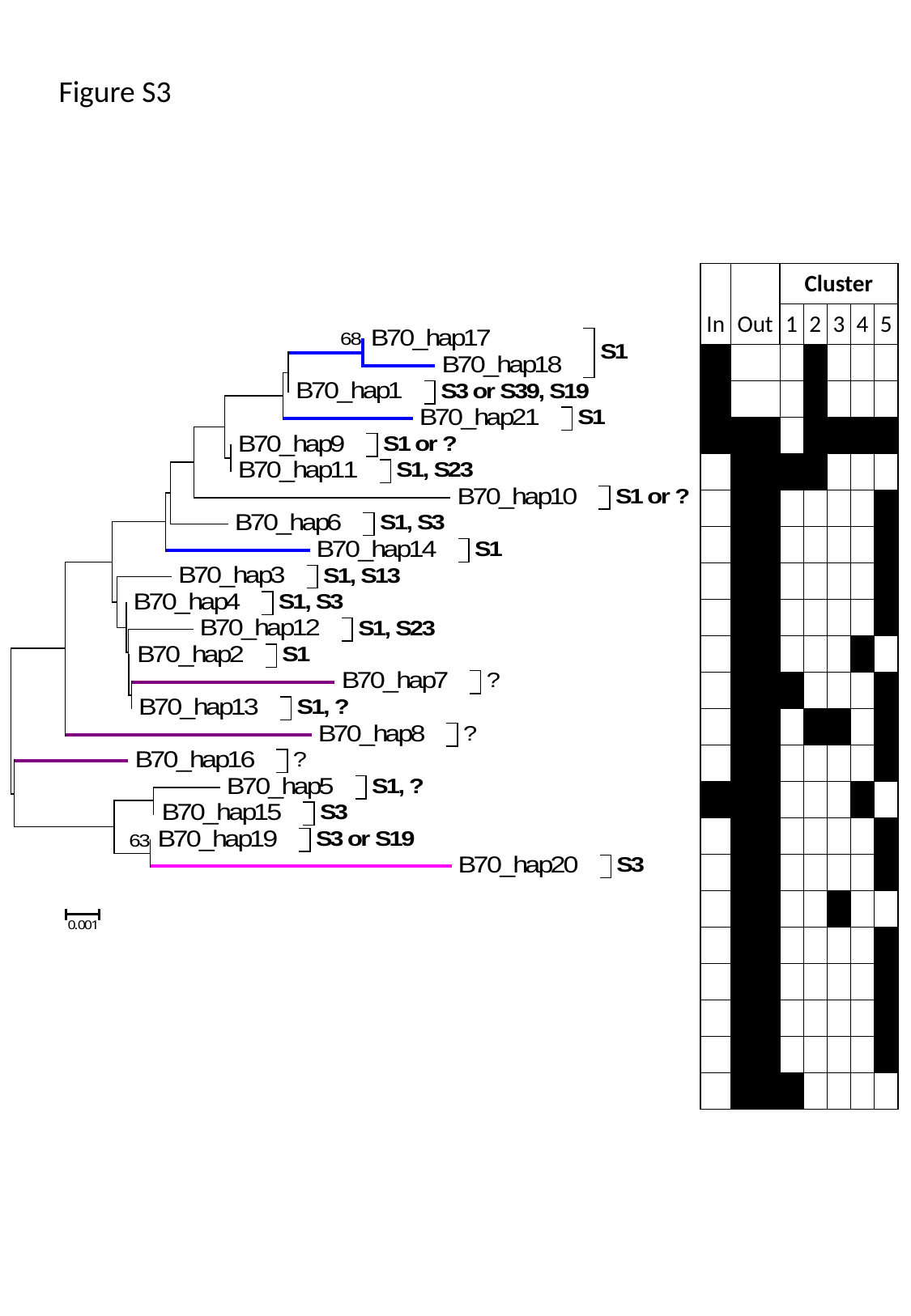

Figure S3
| | | Cluster | | | | |
| --- | --- | --- | --- | --- | --- | --- |
| In | Out | 1 | 2 | 3 | 4 | 5 |
| | | | | | | |
| | | | | | | |
| | | | | | | |
| | | | | | | |
| | | | | | | |
| | | | | | | |
| | | | | | | |
| | | | | | | |
| | | | | | | |
| | | | | | | |
| | | | | | | |
| | | | | | | |
| | | | | | | |
| | | | | | | |
| | | | | | | |
| | | | | | | |
| | | | | | | |
| | | | | | | |
| | | | | | | |
| | | | | | | |
| | | | | | | |

## Slide 4
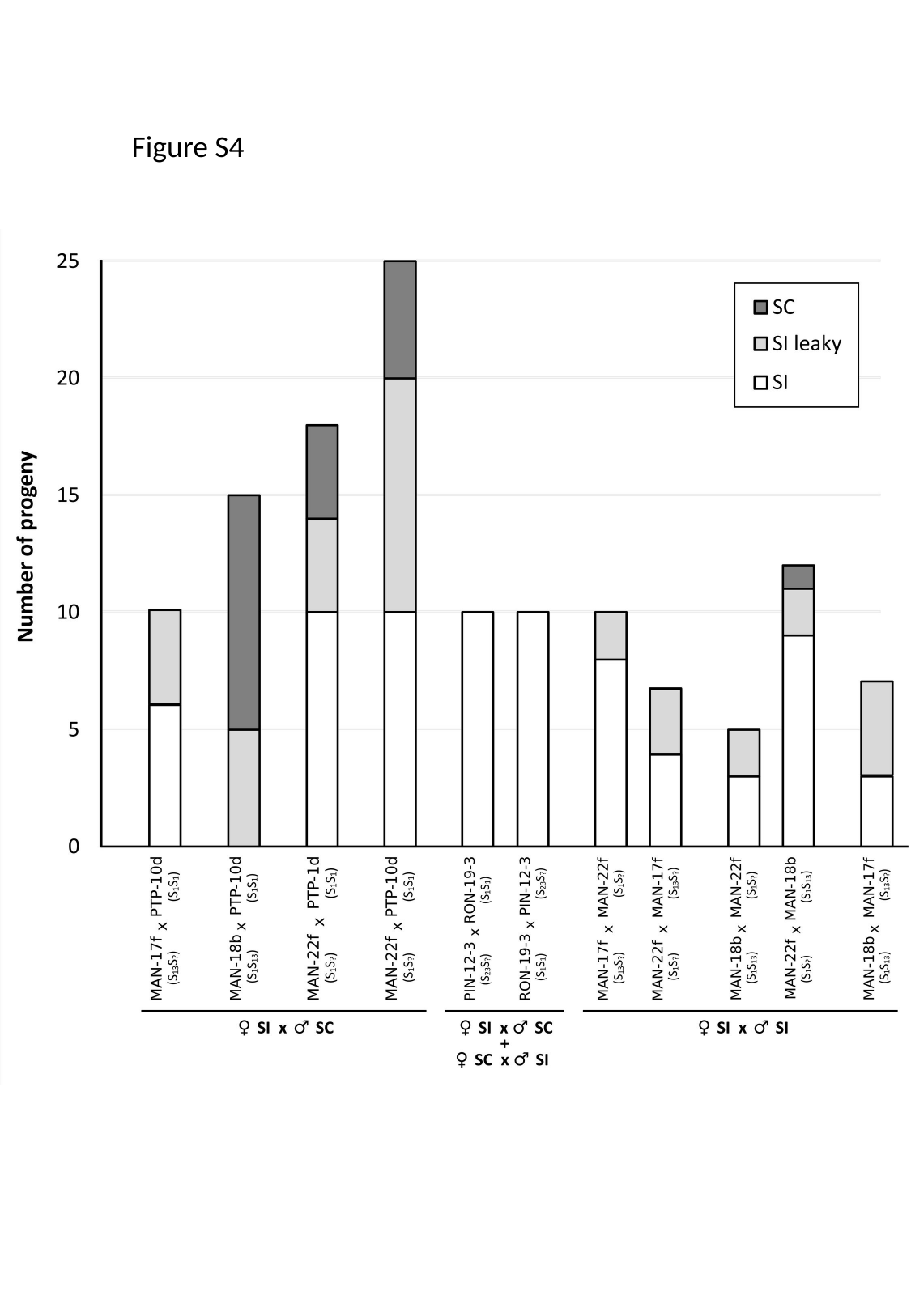

Figure S4

## Slide 5
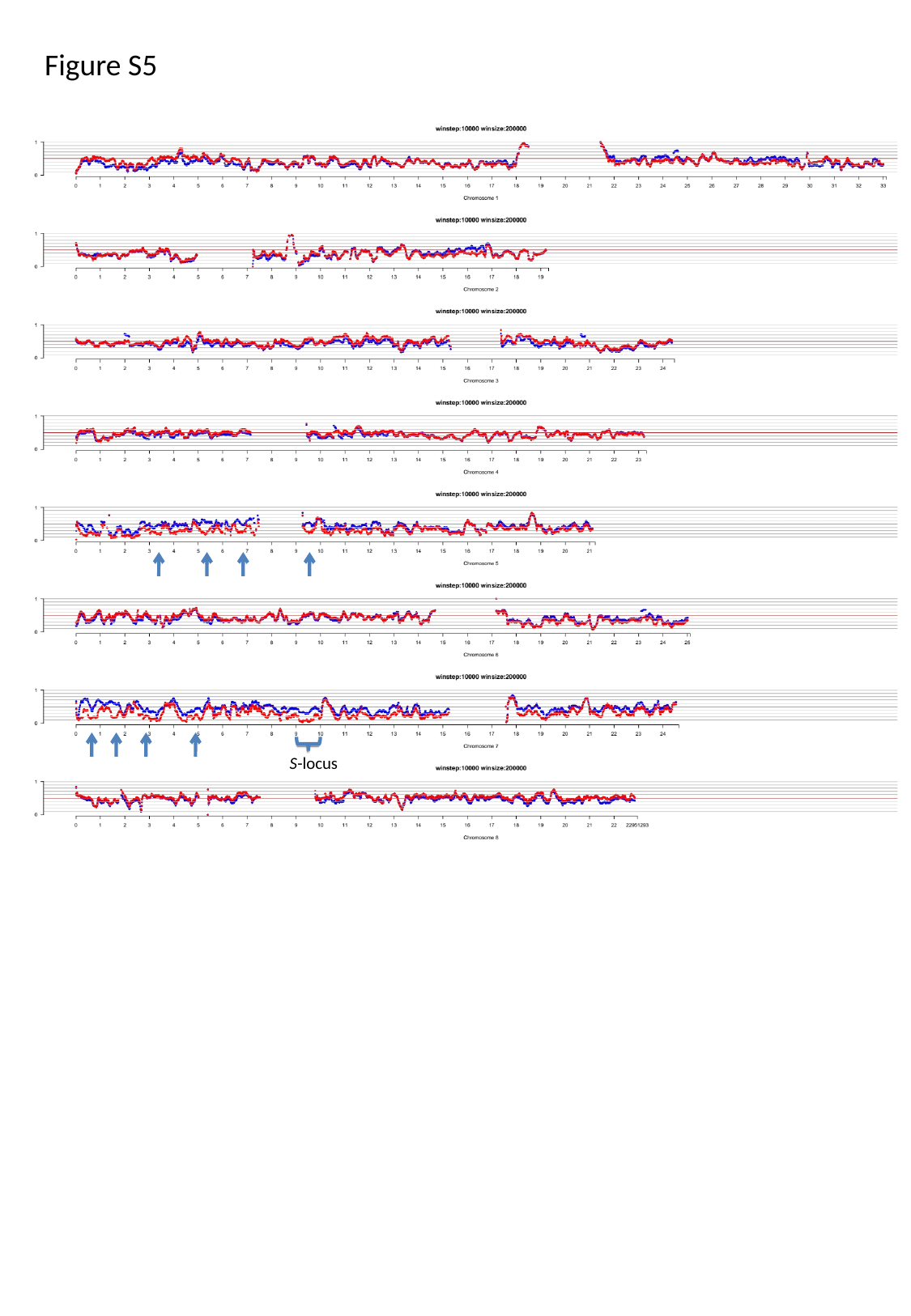

Figure S5
S-locus

## Slide 6
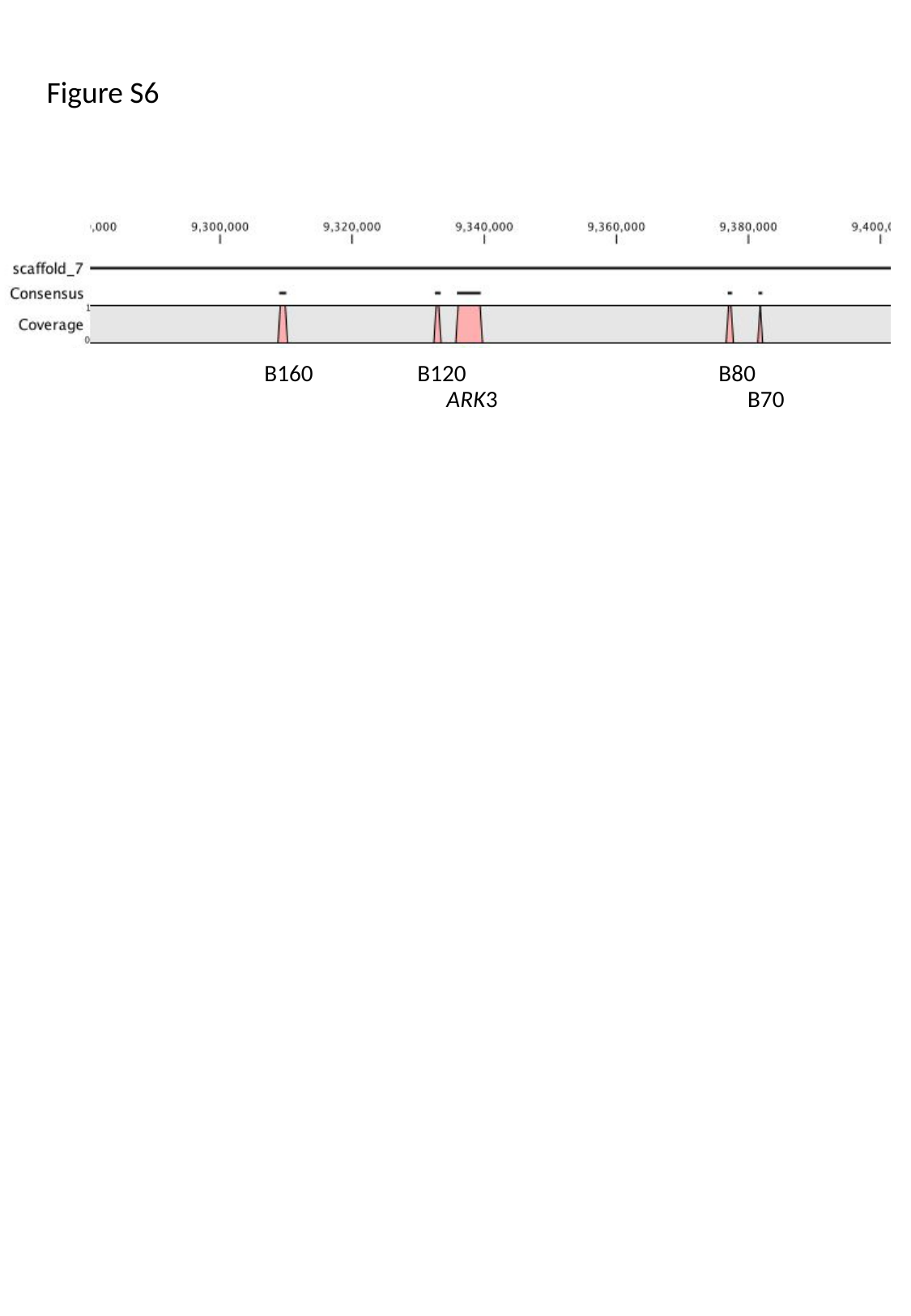

Figure S6
B160
B120
B80
ARK3
B70
